# Combination of E- and NS1-Derived DNA Vaccines: The Immune Response and Protection Elicited in Mice against DENV2

**DOI:** 10.3390/v14071452

**Published:** 2022-06-30

**Authors:** Paolla Beatriz A. Pinto, Tamiris A. C. Barros, Lauro M. Lima, Agatha R. Pacheco, Maysa L. Assis, Bernardo A. S. Pereira, Antônio J. S. Gonçalves, Adriana S. Azevedo, Ana Gisele C. Neves-Ferreira, Simone M. Costa, Ada M. B. Alves

**Affiliations:** 1 Laboratory of Biotechnology and Physiology of Viral Infections, Oswaldo Cruz Institute, Fiocruz, Rio de Janeiro 21040-900, RJ, Brazil; paolla.beatriz@ioc.fiocruz.br (P.B.A.P.); tamiris.azamor@bio.fiocruz.br (T.A.C.B.); lauro.fma@gmail.com (L.M.L.); agatha.pacheco@ioc.fiocruz.br (A.R.P.); maylessis@gmail.com (M.L.A.); baspereira@gmail.com (B.A.S.P.); ajsg@ioc.fiocruz.br (A.J.S.G.); adriana.soares@bio.fiocruz.br (A.S.A.); 2 Laboratory of Toxinology, Oswaldo Cruz Institute, Fiocruz, Rio de Janeiro 21040-900, RJ, Brazil; anag@ioc.fiocruz.br

**Keywords:** DNA vaccine, dengue vaccine, E protein, NS1 protein

## Abstract

The occurrence of dengue disease has increased radically in recent decades. Previously, we constructed the pE1D2 and pcTPANS1 DNA vaccines encoding the DENV2 envelope (E) and non-structural 1 (NS1) proteins, respectively. To decrease the number of plasmids in a tetravalent candidate vaccine, we constructed a bicistronic plasmid, pNS1/E/D2, encoding these two proteins simultaneously. We evaluated the protective immunity induced in mice vaccinated with the pNS1/E/D2 candidate and compared to the responses elicited by immunization with the former vaccines isolated or in combination. We transfected BHK-21 cells with the different plasmids and detected recombinant proteins by immunofluorescence and mass spectrometry assays to confirm antigen expression. BALB/c mice were inoculated with the DNA vaccines followed by a lethal DENV2 challenge. ELISA, PRNT50, and IFN-gamma ELISPOT assays were performed for the investigation of the humoral and cellular responses. We observed the concomitant expression of NS1 and E proteins in pNS1/E/D2-transfected cells. All E-based vaccines induced anti-E and neutralizing antibodies. However, anti-NS1 antibodies were only observed after immunization with the pcTPANS1 administered alone or combined with pE1D2. In contrast, splenocytes from pNS1/E/D2- or pcTPANS1 + pE1D2-vaccinated animals responded to NS1- and E-derived synthetic peptides. All the DNA vaccines conferred protection against DENV2.

## 1. Introduction

Dengue is an acute arboviral infection, considered by the World Health Organization (WHO) as one of the most rapidly emerging diseases globally. About half of the world’s population is at risk of dengue infection in more than 100 countries [1,2], with an estimated 390 million dengue infections occurring every year and 96 million people manifesting the disease [3]. Given the failure to control the spread of the dengue vector, Aedes mosquitoes, as well as the lack of specific treatment, many efforts have been directed towards the development of an effective and protective vaccine [4].

The Dengvaxia (CYD-TDV), developed by Sanofi Pasteur, is the only licensed dengue vaccine, and since 2015 it has been introduced in 20 endemic countries. Dengvaxia is a live recombinant tetravalent dengue vaccine administered in three doses on a 0/6/12-month schedule to individuals between 9 and 45 years old [5,6]. Unfortunately, the vaccine was not as efficient as expected, and there are important safety concerns about it. Long-term follow-up studies suggest that Dengvaxia predisposes dengue seronegative individuals to manifest more severe forms of the disease when later infected with DENV [7,8,9]. These results led the World Health Organization to restrict vaccination only to individuals with a history of previous DENV infection [10]. Therefore, a safe and effective vaccine against dengue remains a public health challenge.

The dengue virus is classified into four antigenically distinct serotypes, DENV1 to DENV4, which share about 70% similarity [11]. Infection with one serotype confers long-lasting protection to it but not to the other serotypes [12]. The DENV genome is a single-stranded positive-sense RNA that codes for a polyprotein, which is processed into three structural (capsid, membrane, and envelope) and seven non-structural proteins (NS1, NS2A, NS2B, NS3, NS4A, NS4B, and NS5) [11].

The E glycoprotein is the main component of the viral surface [13]. Its structure is organized in an ectodomain comprised of three domains (DI, DII, and DIII), a stem region, and a highly hydrophobic transmembrane anchor that allows the protein to be inserted into the virus membrane [14]. The E protein plays a crucial role in different stages of viral infection, such as virus-cell attachment, entry into the cell, the delivery of genetic material into the cell cytoplasm, and viral particle assembly [15,16]. Moreover, this protein is considered the main target for developing dengue vaccines due to its ability to induce neutralizing antibodies (NAb). Many animal models and human cohort studies have shown NAb binding to different domains of the E protein (DI, II, and III) [17]. However, although the contribution of neutralizing antibodies to protection against DENV is of great importance, the assumption of absolute protection provided only by NAb must be carefully rethought. Clinical and experimental data, including the long-term results from the Dengvaxia vaccine, have shown that the protection offered by antibodies needs a fine adjustment of concentration and affinity.

Furthermore, non-neutralizing antibodies can be involved in the phenomenon of antibody-dependent enhancement (ADE) of virus replication, which may be responsible for developing severe forms of the disease [18]. On the other hand, the importance of the cellular immune response in the protection against dengue has been highlighted by several recent reports [19,20,21]. Thus, the combination of different viral antigens may be an important strategy for inducing protective immune responses.

The NS1 is a glycoprotein whose function is not yet fully elucidated, although it is well known that it plays an important role in the DENV replication cycle. It is found inside virus-infected cells, secreted to extracellular medium as hexamers, and associated with plasma cell membranes by a glycosyl–phosphatidylinositol (GPI) link [22,23,24]. This protein induces strong humoral and cellular immune responses, leading to protection [25,26,27,28,29,30,31,32]. In this context, combining the E and NS1 proteins is an attractive strategy for a vaccine against DENV by inducing immune responses that act in different stages of infection.

Previously, our group constructed two DNA vaccines encoding the E, pE1D2 [33], and NS1, pcTPANS1 [29], proteins from DENV2. Both vaccines triggered specific humoral and cellular immune responses that led to protection in mice [29,34,35,36]. The goal of the present work was to evaluate the synergistic effect of E and NS1 antigens in a single plasmid, pNS1/E/D2, or in a combination of pcTPANS1 and pE1D2. The immunogenicity and protection conferred by the DNA vaccines were evaluated in immunocompetent BALB/c mice. The results show that the pNS1/E/D2 induced humoral and cellular responses against the E protein, with NAb and IFN-γ production, while only the T cell response against the NS1 was significant. The combination of the pE1D2 and pcTPANS1 DNA vaccines elicited robust humoral and cellular immune responses towards both E and NS1 proteins and led to complete protection, with no morbidity appearance in animals after the viral challenge. Overall, our results show that the combination of both NS1 and E antigens is a promising approach for developing a DNA vaccine against DENV.

## 2. Materials and Methods

### 2.1. Cells and Dengue Virus

The dengue 2 virus (DENV2), strain New Guinea C (NGC) (GenBank NCBI, M29095, Bethesda, MD, USA), was used for cloning the E and NS1 sequences, as well as for mice challenge assays. The DENV2 44/2 [37] was used for plaque reduction neutralization tests (PRNT_50_). The PRNT_50_ was carried out in Vero cells maintained in 199 medium with Earle’s salts (E199, Sigma, St. Louis, MO, USA) containing 2.5% *v*/*v* sodium bicarbonate, supplemented with 5% fetal bovine serum (FBS, Invitrogen, Waltham, MA, USA) and gentamicin (0.04 mg/mL, Sigma) at 37 °C with 5% CO_2_. For the analysis of in vitro expression of recombinant E and NS1 proteins, baby hamster kidney fibroblasts (BHK-21) were maintained in English Dulbecco’s Modified Eagle Medium (DMEM, Sigma) added with 5% FBS (Invitrogen) at 37 °C with 5% CO_2_.

### 2.2. Previous DNA Vaccines

The DNA vaccines pE1D2, pcTPANS1, and pcTPA were previously described [29,33]. Briefly, the pE1D2 plasmid encodes the ectodomain (domains I, II, and III) of the DENV2 E protein, while pcTPANS1 contains the full-length DENV2 *ns1* gene. In both constructions, genes were fused to the sequence coding the human tissue plasminogen activator (t-PA) signal peptide under the control of the cytomegalovirus (CMV) promoter region. The negative control pcTPA was derived from the commercial vector pcDNA3 (Invitrogen) and contains only the t-PA signal peptide sequence.

### 2.3. Construction of the Plasmid pNS1/E/D2

The strategy for cloning the sequence coding the ectodomain of the E protein and the *ns1* gene in a single plasmid was to synthesize the NS1 expression cassette and subclone it into the pE1D2 plasmid by substituting the neomycin resistance gene present in this plasmid. The NS1 expression cassette was drawn to contain part of the simian virus 40 (SV40) promoter sequence (from nucleotides 2090 to 2115 in the pcDNA3), the t-PA signal sequence, the full-length DENV2 *ns1* gene (nucleotides 2422 to 3477 in the NGC DENV2 genome), and the SV40 poly A sequence (nucleotides 2933 to 2965 in the pcDNA3). This NS1 cassette was synthesized by GenScript (GenScript Biotech, Piscataway, NJ, USA) and cloned into the pUC57 vector, resulting in the plasmid named prSV40-NS1. To replace the neomycin gene present in pE1D2 by the NS1 sequence, both plasmids (pE1D2 and prSV40-NS1) were restricted with *Sma* I and *Csp*45 I enzymes (Promega, Madison, WI, USA) and electrophoresed on a 1% agarose gel. Fragments with higher and lower molecular weights, 4.3 Kb from pE1D2 and 1.3 Kb from prSV40-NS1, were recovered with glass beads Geneclean (MP Biomedicals, Santa Ana, CA, USA ) and ligated with T4 DNA Ligase Cloning Qualified (Promega). *Escherichia coli*, DH5-α strain, was transformed with the recombinant plasmids. Positive clones were screened by restriction mapping and confirmed by sequencing (ABI PRISM dye terminator, Applied Biosystems, Waltham, MA, USA), performed by the DNA Sequencing Genomic Platform (IOC-Fiocruz, Rio de Janeiro, RJ, Brazil). The resulting construct was named pNS1/E/D2. The schematic representation of the cloning strategy is described in Figure S1.

### 2.4. DNA Vaccine Purification

Large-scale preparations of all the DNA vaccines and the pcTPA control plasmid were produced from transformed *E. coli* DH5α, grown in TB medium containing ampicillin (100 mg/mL). Purification was performed by alkaline lysis and QIAGEN Endofree Plasmid Giga Kit (QIAGEN, Hilden, NRW, Germany), according to the manufacturer’s instructions. The DNAs were quantified in a spectrophotometer by measuring absorbance at 260 nm. The concentration and integrity of all plasmids were confirmed by agarose gel electrophoresis. Plasmids were suspended in sterile water and stored at −20 °C until use.

### 2.5. Transfection of BHK Cells and Detection of the Recombinant Proteins

BHK-21 cells were transiently transfected with the isolated DNA vaccines pNS1/E/D2, pE1D2, or pcTPANS1; the combination of pE1D2 and pcTPANS1; or with the pcTPA negative control, before E and NS1 detection by immunofluorescence assay (IFA), flow cytometry, or mass spectrometry.

#### 2.5.1. Immunofluorescence Assay

Cells were plated overnight on chamber slides (LabTek, Nunc, Roskilde, Denmark), 2 × 10^4^ cells/well, with Opti-MEM medium (Invitrogen). The next day, cells were transfected with 0.4 µg of one of the recombinant plasmids using lipofectamine (Invitrogen), according to the manufacturer’s recommendations. After 24 h, cells were fixed with 4% paraformaldehyde in 0.1 M sodium phosphate buffer (PB) for 15 min, permeabilized with 0.6% saponin in PB for 10 min, and blocked with 1% bovine serum albumin (BSA) and 0.2% saponin in PB for 15 min at room temperature. The E protein was detected with the monoclonal anti-DENV2 3H5 antibody (ATCC). To detect the NS1 protein, cells were incubated with anti-NS1 polyclonal antibodies produced in rabbits inoculated with recombinant DENV2 NS1 protein expressed in the *Drosophila* S2 cell line (kindly provided by Dr. Beth-Ann Coller, Merck, Rahway, NJ, USA). Cells were incubated for 1 h at 37 °C with primary antibodies, washed with PB, and incubated for another 1 h at 37 °C with goat anti-rabbit IgG conjugated to Alexa Fluor 488 (AF 488, Molecular Probes, Eugene, OR, USA) and/or with goat anti-mouse IgG conjugated to Alexa Fluor 546 (AF 546, Molecular Probes). Cells were then washed with PB, and slides were mounted with Vectashield medium (Vector Laboratories Inc., Newark, CA, USA). Cells were visualized in the Nikon H550S fluorescence microscope.

#### 2.5.2. Flow Cytometry

BHK-21 cells were seeded in T75 culture flasks, 2 × 10^6^ cells/flask, with Opti-MEM medium (Invitrogen). The next day, cells were transfected with 12 µg of one of the recombinant plasmids using lipofectamine (Invitrogen), according to the manufacturer’s recommendations. After 24 h, cells were dissociated with Trypsin EDTA, washed in staining buffer (PBS, 2% FCS, 2 mM EDTA, 5 × 10^−5^ Mβ-mercaptoethanol), fixed, and permeabilized using Cytofix/Cytoperm solution (BD Biosciences, Franklin Lakes, NJ, USA), following the manufacturer’s instructions. Intracellular staining of E and NS1 proteins was conducted using the same primary and secondary antibodies described in item 2.5.1. All antibodies were diluted in 20 μL staining buffer, and the cells were incubated for 30 min at 4 °C. Fifteen thousand cells were acquired in a CytoFLEX flow cytometer (Beckman Coulter, Pasadena, CA, USA), and data were analyzed using CytExpert software 2.0 (Beckman Coulter).

#### 2.5.3. Mass Spectrometry

BHK-21 cells were seeded in T25 culture flasks, 3 × 10^5^ cells/flask, with Opti-MEM medium (Invitrogen). The next day, cells were transfected with 8 µg of one of the recombinant plasmids using lipofectamine (Invitrogen), according to the manufacturer’s recommendations. After 24 h, the cells were washed three times with serum-free DMEM to remove serum protein contaminants from the cell culture medium and were then kept in the serum-free medium for 8 h at 37 °C with 5% CO_2_. The culture supernatants were then collected (2 mL), centrifuged, and precipitated in 10% trichloroacetic acid and 0.1% Triton X-100 for 2 h at 4 °C. The protein precipitate was washed twice with 90% acetone, dried at room temperature, and solubilized in 50 µL electrophoresis sample buffer. Samples (25 µL) were analyzed by polyacrylamide gel electrophoresis (12% T), in the presence of sodium dodecyl sulfate (SDS) and dithiothreitol (DTT), and the gels were stained with Coomassie blue R250. Two bands were cut in the region bounded by the 45 and the 66 kDa molecular mass standards in each gel lane. Each gel band was then individually submitted to in-gel digestion with trypsin, as previously described [38]. After gel extraction, the peptides were dried in a vacuum centrifuge concentrator and suspended in 12 µL of 1% formic acid. They were then directly analyzed by MS/MS using an Easy-nLC 1200 UHPLC system coupled to a QExactive Plus mass spectrometer with an nESI source (all from Thermo Fisher Scientific, Waltham, MA, USA). The tryptic peptides in 1% (*v*/*v*) formic acid were injected (4 µL) on a trap column (Acclaim PepMap™ 100, 3 µm particle size, 75 µm inner diameter, 100 Å pore size, and 2 cm long, nanoViper) at a flow rate of 2 µL/min. Peptide fractionation was carried out on a C_18_ Micro Pillar Array Column (µPAC™, 200 cm length) (COL-nano200G1B, PharmaFluidics, Thermo Fisher Scientific, Waltham, MA, USA), operating at a flow rate of 400 nL/min. The column outlet was connected to a fused silica capillary (20 µm inner diameter, 7 cm long), followed by a liquid junction and a PicoTip™ emitter (20 µm inner diameter, 10 µm tip, 12 cm long, no coating, New Objective, Littleton, MA, USA). The following mobile phases were used: (A) 0.1% formic acid (*v*/*v*) in water; (B) 0.1% formic acid (*v*/*v*) in 80% acetonitrile (*v*/*v*). The linear elution gradient used was 1–50% B in 60 min. Phase B concentration was then raised to 97% in 10 s, and the column was kept under this last elution condition for an additional 5 min and 50 s before column re-equilibration for 25 min (91 min total method duration). The mass spectrometer was operated in the data-dependent mode (DDA), using a top 12 method as previously described [39], except for dynamic exclusion (15 s) and column temperature (50 °C). Data were acquired after a single injection of each gel fraction using the Xcalibur software (Version 3.0.63, Thermo Fisher Scientific, Waltham, MA, USA). To minimize cross-contamination, two blank injections were run before analyzing each biological replicate (i.e., different gel lanes). Proteins were identified by the peptide-spectrum matching (PSM) approach, using the PatternLab for Proteomics V (PLV) search and filtering algorithm [40] and a database containing the following sequences: (1) t-PA signal peptide + NS1 protein (DENV2-New Guinea strain. Both sequences were separated by the two amino acid residues coded by the restriction site used for cloning); (2) t-PA signal peptide + the first 80% amino acid residues of the envelope protein (DENV2–New Guinea strain. Both sequences were separated by the two amino acid residues coded by the restriction site used for cloning); (3) 123 protein sequences corresponding to the contaminant library of PLV software. After eliminating subset sequences (considering 100% identity) in PLV, 20,291 target sequences were counted. PLV automatically reversed all target sequences to generate the final target-decoy database. The following criteria were used to search the database: (a) semi-tryptic specificity; (b) ≤2 missed cleavages allowed; (c) carbamidomethyl cysteine (+57.02146 Da) as fixed modification; (d) oxidized methionine (+15.9949) as variable modification; (e) no more than two variable modifications per peptide; and (f) search tolerance ≤ 10 ppm from the error mean. The search results corresponding to the two fractions of each gel lane were filtered together to achieve a ≤1% false discovery rate (FDR) at the spectral, peptide, and protein levels.

### 2.6. Mice Immunization and Challenge with DENV2

In vivo experiments were conducted in compliance with ethical principles in animal experimentation stated in the Brazilian College of Animal Experimentation and approved by the Animal Use Ethical Committee of Oswaldo Cruz Institute in Oswaldo Cruz Foundation (CEUA-IOC approval ID: L039/2015 and L022/2019).

Groups of male BALB/c mice, specific pathogens free (SPF), 4 weeks old, were purchased from the Multidisciplinary Center for Biological Investigations (CEMIB, UNICAMP, Campinas, SP, Brazil). Animals were inoculated by the intramuscular route (i.m.) with 50 μg of plasmids diluted in 50 μL of phosphate buffer saline (PBS) in each tibialis posterior muscle. Each animal group received two doses of one of the following DNA vaccines: pNS1/E/D2, pE1D2, or pcTPANS1 individually (100 μg of DNA) or a mixture of the two plasmids pE1D2 + pcTPANS1 (50 μg of each plasmid). The DNA vaccines were administered at a two-week interval, and immunized mice were euthanized two or four weeks after the second DNA dose. Blood was collected through heart puncture, and sera were stored at −70 °C until use in ELISA and PRNT assays. For E- and NS1-specific antibody response analysis, mice were previously bled by retro-orbital puncture just before immunization (pre-immune sera). Spleens were also harvested from immunized mice two or four weeks after the second DNA dose, and splenocytes were used for IFN-γ-ELISPOT assays.

For protection assessment, immunized mice were challenged by the intracerebral (i.c.) route two weeks after the second DNA dose with 30 μL of a neuroadapted NGC DENV2, which corresponds to 3.5 log_10_ PFU/mL and approximately 40 LD_50_, diluted in serum-free E199 medium. Non-immunized mice were also inoculated with DENV2 as virus infection controls. After the DENV2 challenge, animals were monitored for 3 weeks regarding mortality and morbidity rates. Mice inoculated with pcTPA were added as a control group for immunogenicity and protection tests.

To assess morbidity, clinical signs such as leg paralysis and the commitment of the spinal column were recorded according to an arbitrary scale ranging from 0 to 4: 0 = absence of clinical signs; 1 = paralysis in one leg or alteration of the spinal column; 2 = severe paralysis in one leg and alterations of the spinal column or severe paralysis on both hind legs; 3 = severe paralysis in the two hind legs and alterations of the spinal column; and 4 = death. After 21 days, animals were bled by cardiac puncture and euthanized.

**Figure 1:**
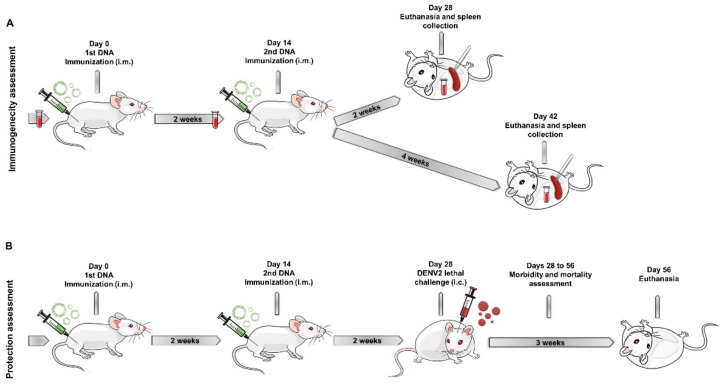
Schematic timeline representation of the in vivo experiments**.** BALB/c mice were inoculated twice in the tibialis muscle with the DNA vaccines. Immunogenicity tests (**A**) were conducted with blood and splenocytes. Blood was collected prior to immunization, before the second DNA dose and 2 or 4 weeks after the second dose, for antibody analysis. The spleen was harvested 2 or 4 weeks after the second DNA dose to analyze the cellular immune response. For protection tests, immunized animals were challenged intracerebrally with a lethal dose of a neuroadapted DENV2. Mortality and morbidity rates were recorded daily for 3 weeks after challenge (**B**).

Figure 1 shows a schematic representation of immunogenicity (Figure 1A) and protection assessment in mice (Figure 1B).

### 2.7. Detection of Antibodies against E and NS1 Proteins

Mouse sera were tested for the presence of specific anti-E or anti-NS1 antibodies by ELISA. Recombinant DENV2 EDIII and NS1 proteins were used as solid-phase bound antigens. The domain III of the E protein (EDIII) was expressed in *E. coli* and kindly given by Dr. Luís Carlos Ferreira (USP, São Paulo, SP, Brazil), while the recombinant NS1 protein was expressed in S2 cells and gently provided by Dr. Beth-Ann Coller (Merck Sharp and Dohme Corp., Rahway, NJ, USA). Both E and NS1 were also heat-treated at 100 °C for 10 min for ELISA assays using denatured proteins to investigate the production of antibodies against conformational epitopes of these proteins. Pre-immune or immune sera were collected two weeks after the second DNA dose.

MaxiSorp plates (Nunc) were incubated with the recombinant proteins (0.2 µg of EDIII or 0.1 µg of NS1/well) for 1 h at 37 °C, and wells were subsequently blocked with 2% g/L skim milk in 0.05% *v*/*v* Tween-20-PBS (PBST) at 4 °C overnight. The next day, pre-immune or immune sera collected two or four weeks after the second DNA dose were five-fold serially diluted, added in duplicates to ELISA plates previously washed with PBST, and incubated for 1 h at 37 °C. Plates were then washed with PBST, incubated for 1 h at 37 °C with goat anti-mouse IgG conjugated to horseradish peroxidase (Southern Biotechnology, Birmingham, AL, USA) diluted in PBS, and washed again with PBST. Reactions were measured at A_490mm_ in a microplate reader (Spectra Max 190, Molecular Devices, San José, CA, USA) with ortho-phenylenediamine dihydrochloride (Sigma) and H_2_O_2_ as substrate and stopped with 9 N H_2_SO_4_. Titres were established as the reciprocal of serum dilution, which produced an absorbance above that of the respective pre-immune serum. 

### 2.8. Plaque Reduction Neutralization Test (PRNT_50_)

The PRNT_50_ assays were performed on monolayers of Vero cells in 96-well plates. Before the experiment, sera samples were heated to 56 °C for 30 min to inactivate the complement system. Shortly, cells were seeded at a density of 1.0 × 10^5^ cells/well in E199 medium with 5% FBS and cultured at 37 °C with 5% CO_2_ for 24 h. The next day, serum samples were two-fold serially diluted (from 1:20 to 1:1260) in 60 µL of E199 medium; mixed with 60 µL of DENV2 44/2, containing approximately 30 PFU; and then incubated for 1 h at 37 °C. Media from the 96-well plates were discarded, 100 µL of the serum/virus mixture was added in duplicates, and plates were incubated for 1 h at 37 °C with 5% CO_2_. The supernatant of each well was then discarded, and 150 µL E199 medium with 5% FBS and 2% p/v carboxymethylcellulose (CMC, Sigma) were added. The plates were incubated for 5 days at 37 °C with 5% CO_2_. After this period, the cell monolayers were fixed with 10% formalin solution for 24 h and stained with 0.02% crystal violet for 30 min. Neutralizing antibody titers were expressed by 50% plaque reduction (PRNT_50_).

### 2.9. Interferon Gamma ELISPOT Assay

Groups of BALB/c mice (*n* = 5) were euthanized two or four weeks after immunization, and splenocytes were used in IFN-γ ELISPOT test. The assay was performed with the IFN-γ ELISPOT mouse set (BD Biosciences) according to the manufacturer’s instructions. For tests performed two weeks after the second DNA dose, cells were stimulated with synthetic peptides contained in the E (*^331^*SPCKIPFEI*^339^*) or NS1 (*^265^*AGPWHLGKL*^273^*) proteins, both described as specific to the CD8^+^ T cell [41,42]. Splenocytes isolated four weeks after the second DNA dose were stimulated with a positive E- or NS1- derived peptide pool, previously screened and able to induce CD4^+^ and CD8^+^ activation [36].

Briefly, 96-well ELISPOT plates were coated overnight at 4 °C with anti-IFN-γ capture monoclonal antibody (100 µL/well, diluted in PBS). The next day, plates were washed with PBS and blocked with RPMI-1640 medium (Sigma) supplemented with 10% SFB for 2 h at 37 °C. Splenocytes were added in triplicates (5.0 × 10^5^ cells/well) in RPMI-1640 medium containing 10% SFB, followed by the addition of the peptide suspension (2 µg/well). Non-stimulated and concanavalin A (Con A, 2 µg/well) stimulated cells were used as negative and positive controls, respectively. Plates were maintained for 18 h at 37 °C with 5% CO_2_. After this period, the cells were discarded, plates were washed twice with distilled water and then with PBST, followed by incubation for 2 h at 37 °C with anti-IFN-γ biotinylated detection antibody diluted in PBS. Plates were rewashed with PBST and incubated for 1 h with streptavidin-horseradish peroxidase (HRP) conjugate. Finally, plates were washed with PBST and revealed with the addition of the AEC substrate set (BD/Pharmingen) and hydrogen peroxide (Sigma) for 20 min at room temperature. The reaction was stopped by washing plates with distilled water. Spots were counted in the automated immunospot reader (Cellular Technology Ltd., Shaker Heights, OH, USA) at the ELISPOT Multi-User Platform (Fiocruz).

### 2.10. Statistics

Statistical differences were assessed using the GraphPad Prism v9.0 program (San Diego, CA, USA), with a minimum level of significance of 95%. The non-parametric Mann Whitney test was performed to analyze the statistical differences observed in ELISA and ELISPOT, and the degree of morbidity after virus challenge. The survival and morbidity rates were evaluated using the Log-Rank (Mantel–Cox test) statistical test.

## 3. Results

### 3.1. Detection of the Recombinant E and NS1 Proteins in Cells Transfected with the DNA Vaccines

**Figure 2:**
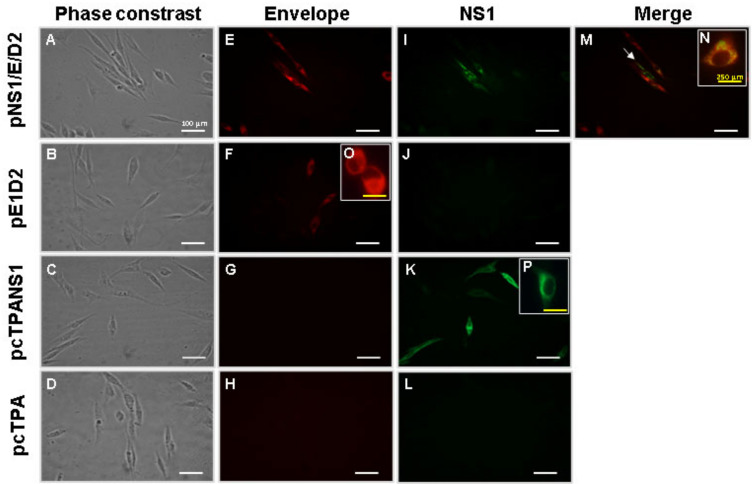
In vitro expression of the E and NS1 proteins in cells transfected with the different DNA vaccines. BHK-21 cells were transiently transfected with pNS1/E/D2, pE1D2, pcTPANS1, or the negative control pcTPA. The E protein was detected with the mouse 3H5 monoclonal antibody (against domain III) and goat anti-mouse IgG Alexa Fluor 546 antibody (red fluorescence). The NS1 protein was detected with rabbit anti-NS1 polyclonal serum, followed by incubation with goat anti-rabbit IgG Alexa Fluor 488 antibody (green fluorescence). Phase contrast (**A**–**D**). Cells were incubated with anti-EDIII of the E protein (**E**–**H**,**O**) and anti-NS1 (**I**–**L**,**P**) antibodies. Images merge (**M**,**N**). Cells were analyzed by fluorescence microscopy at 40× (**A**–**M**) or 100× (**N**–**P**) magnifications. The white arrow indicates the pNS1/E/D2-transfected cell expressing only the NS1 protein. Scale white bar, 100 μm; scale yellow bar, 250 μm.

To evaluate the ability of the new DNA vaccine construct, pNS1/E/D2, to mediate the expression of both E and NS1 proteins, BHK-21 cells were transfected with this plasmid, as well as with the positive E (pE1D2) and NS1 (pcTPANS1), and negative (pcTPA) controls. The expression of the recombinant proteins was assessed by indirect immunofluorescence assay (IFA), using specific anti-E and anti-NS1 antibodies followed by incubation with secondary antibodies conjugated to AF 546 and AF 488 for red and green fluorescence, respectively. Cells transfected with the pNS1/E/D2 DNA vaccine showed red and green fluorescence specific to E and NS1 proteins, respectively (Figure 2E,I). The merge of fluorescence images revealed pNS1/E/D2-transfected cells expressing each of the proteins separately and concomitantly (Figure 2M,N). The E or NS1 proteins were detected after transfection with pE1D2 (Figure 2F,O) or pcTPANS1 (Figure 2K,P), respectively. No cross-reactivity between anti-E and anti-NS1 antibodies was observed, as well as with the secondary antibodies, thus validating the specific antibody labeling for the two recombinant proteins (Figure 2G,J). As expected, no positivity was observed in cells transfected with the pcTPA negative control (Figure 2H,L).

**Figure 3:**
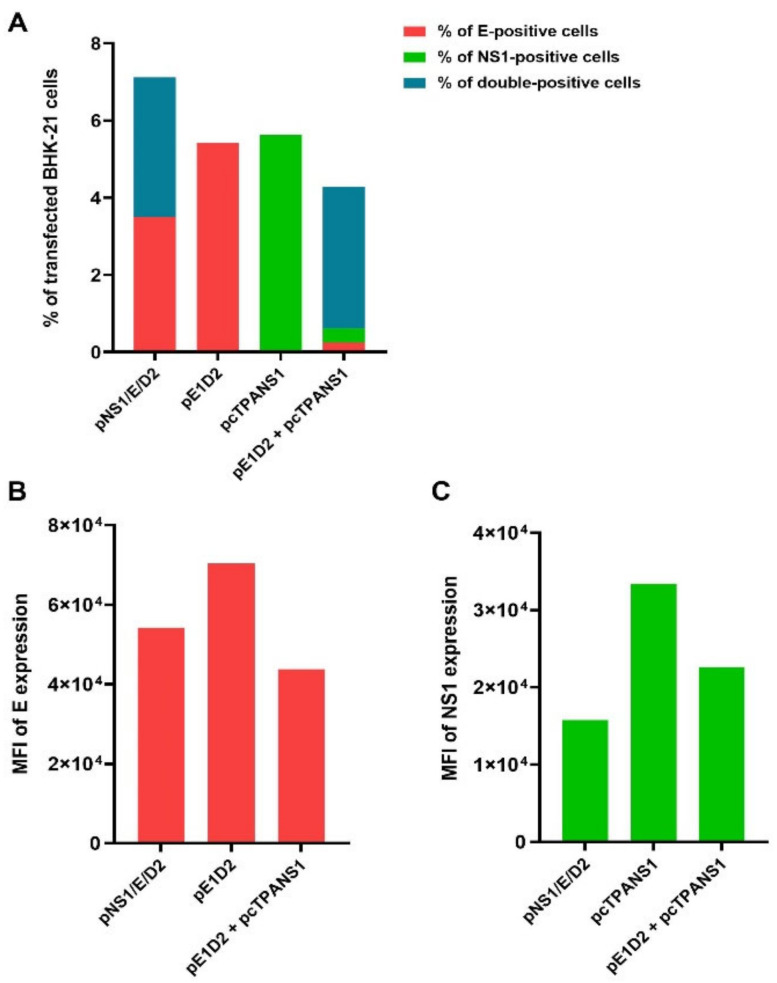
Quantification of in vitro expression of the E and NS1 proteins in cells transfected with the different DNA vaccines. Flow cytometry analysis showing E- and NS1-positive frequencies in BHK-21 cells transfected with the control pcTPA or with the DNA vaccines pNS1/E/D2, pE1D2, pcTPANS1, and pE1D2 + pcTPANS1 (**A**), and the mean of fluorescence intensity (MFI) for E (**B**) and NS1 staining (**C**). Transient transfection was carried out with lipofectamine reagent. After the 24h-transfection, cells were harvested and stained with mouse monoclonal anti-E (3H5) antibody and rabbit polyclonal anti-NS1 antibodies, followed by incubation with goat anti-mouse IgG conjugated to Alexa Fluor 546 and goat anti-rabbit IgG conjugated to Alexa Fluor 488.

The flow cytometry analysis of transfected BHK-21 cells was used as a second detection technique to quantify the number of E- and NS1-positive cells and analyze the amount of protein expressed by these cells based on the fluorescence intensity (MFI). BHK-21 cells were transfected as above, and E and NS1 proteins were detected after intracellular staining with the same antibodies. The percentages of positive E and NS1-cell populations and MFI for each fluorochrome are shown in Figure 3. The gating strategy for flow cytometry analysis is shown in the supplementary material (Figure S2).

Results from the flow cytometry confirmed the positive detection of E and NS1 proteins in pNS1/E/D2-transfected cells. Transfection with the pNS1/E/D2 led to cells expressing the E protein equally, alone (3.50%), or in combination with the NS1 (3.65%), while no cells expressing only the NS1 were detected (Figure 3A). On the other hand, transfection with the mixture of pE1D2 + pcTPANS1 led most cells to simultaneously express both E and NS1 proteins (3.67%) compared to E or NS1 individually (0.36% and 0.25%, respectively). The MFI numbers were higher in the cells transfected only with pE1D2 or pcTPANS1 (Figure 3B,C), suggesting that the expression of each protein individually is more efficient than in combination. However, the results indicate that the newly constructed pNS1/E/D2 DNA vaccine was able to mediate the expression of the E and NS1 proteins, confirming the functionality of the two promotor regions that control the independent expression of both proteins.

### 3.2. Identification and Quantification of Secreted E and NS1 Proteins in the Supernatant of Cells Transfected with the DNA Vaccines

Antigen secretion after DNA vaccination is an important component for triggering and activating an efficient immune response. In our DNA constructs, E and NS1 genes were fused to the t-PA signal peptide for the secretion of the antigenic proteins. To access the DNA vaccine capacity to secrete both E and NS1 proteins, mass spectrometric analysis was performed using the supernatant of BHK-21 cells transfected with the DNA vaccines. The samples were fractionated by SDS-PAGE, and the gel regions spanning 45 to 66 kDa were processed for protein identification by nLC-MS/MS (Figure S3).

The E and NS1 proteins were identified by MS/MS in the supernatant of BHK-21 cells transfected with the pNS1/E/D2 plasmid with high confidence: the envelope protein was identified by 30 unique peptides (62 unique spectral counts), while 25 unique peptides were sequenced for NS1 (58 unique spectral counts). Relative abundances of both proteins, estimated based on the normalized spectral abundance factor (NSAF), were similar when pNS1/E/D2 was used to transfect the cells (Table 1). Comparable amounts of the envelope protein were detected in the supernatants of cells transfected with the following DNA vaccine formulations: pNS1/E/D2 (62 spectral counts), pE1D2 (85 spectral counts), and the mixture pE1D2 and pcTPANS1 (71 spectral counts). On the other hand, the abundance of NS1 protein was about one order of magnitude higher when the cells were transfected with pcTPANS1 alone (627 spectral counts) or in combination with pE1D2 (484 spectral counts) in comparison to pNS1/E/D2 (58 spectral counts). Figure S4 shows detailed information on dengue virus proteins identified by MS/MS in the supernatant of BHK-21 cells transfected with different DNA vaccines. In addition to virus proteins, the mass spectrometry analysis of all supernatants identified several contaminant proteins, such as keratins and bovine serum albumin (Table S1).

IFA, flow cytometry, and mass spectrometry analyses showed that the pNS1/E/D2 vaccine can mediate the expression of E and NS1 antigens in vitro, and these proteins were successfully secreted into the extracellular space. However, the expression of NS1 protein was less effective when compared to the expression mediated by the pcTPANS1.

### 3.3. Antibody Response against the E Protein Generated by the DNA Vaccines

**Figure 4:**
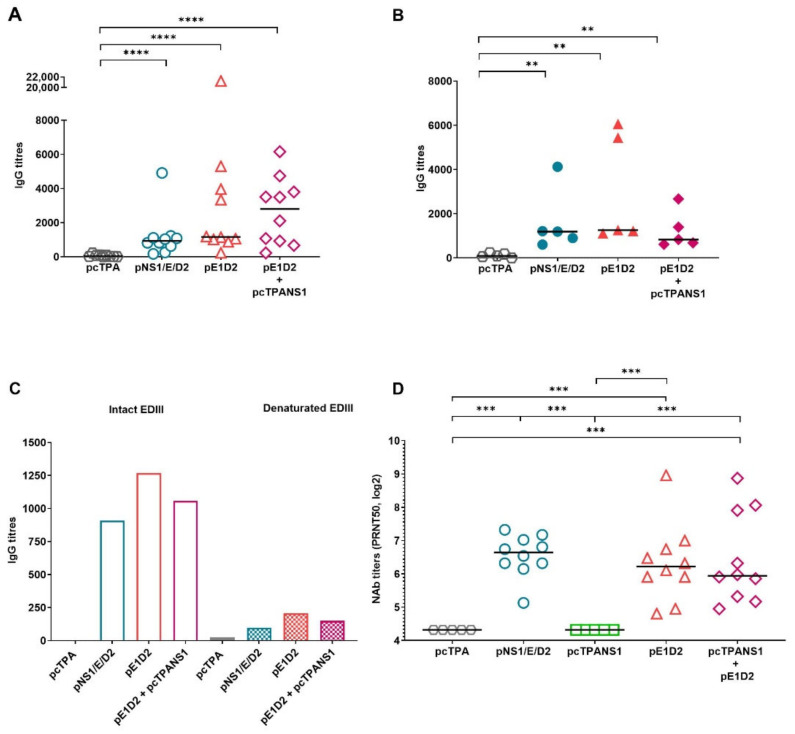
Antibody response directed to the E protein in serum samples of BALB/c mice immunized with E-derived DNA vaccines**.** Antibody titration against the EDIII protein by ELISA (**A**–**C**) and PRNT_50_ (**D**) using serum samples from mice immunized with pNS1/E/D2, pcTPANS1, and pE1D2, alone or in a mixture of pE1D2 + pcTPANS1 (*n* = 5–10), and the control group inoculated with pcTPA (*n* = 5–10). Serum samples date from 2 (**A**,**C**,**D**) or 4 weeks (**B**) after the second DNA dose. Samples from each animal were individually tested in duplicates, and bars represent the median of the obtained values. All ELISA assays were performed using the intact EDIII protein, except in (**C**), in which both intact and heat-denatured protein were used for testing pooled serum samples of 5 mice per group (data shown are the median of the obtained values). The NAb values from the pcTPA and pcTPANS1 groups were shown as the minimum dilution of the PRNT test (1:20) in a log2 scale. Asterisks in (**A**,**B**,**D**) indicate statistically significant differences between experimental groups using the non-parametric two-tailed Mann-Whitney test (** *p* < 0.01; *** *p* < 0.001; and **** *p* < 0.0001). Data obtained with sera collected 2 weeks after the last immunization are representative of two independent experiments (*n* = 4–5), except for the pcTPA and pcTPANS1 groups in (**D**).

The humoral immune response directed to the E protein was assessed by ELISA using the EDIII protein as a solid-phase antigen, and mouse sera were collected two or four weeks after the second DNA dose. All the E-based DNA vaccines induced significant anti-EDIII antibodies two weeks after the last immunization compared to control animals inoculated with the pcTPA plasmid (Figure 4A). Anti-EDIII IgG titers remain detectable and statistically significant in all animal groups four weeks after immunization with the E-derived vaccines (Figure 4B).

The EDIII protein was also heat-denatured to assess the conservation status of the recombinant protein in ELISA assays. There is a notable decrease in IgG titers after the denaturation of EDIII, supporting the notion that antibodies raised by DNA immunization recognize mainly conformational epitopes of the E protein (Figure 4C).

In addition, serum samples obtained two weeks after the last immunization were tested for their ability to neutralize the DENV2 infection in VERO cells by PRNT_50_ assays. Immunization with pNS1/E/D2, pE1D2, or pE1D2 + pcTPANS1 induced significant production of NAb titers compared to the control group pcTPA (*p* < 0.001) (Figure 4D). We did not observe statistically significant differences in NAb titers elicited by the E-derived DNA vaccines, pNS1/E/D2, pE1D2, and pE1D2 + pcTPANS1, albeit one mouse in the pE1D2-immunized group and three animals vaccinated with the mixture pE1D2 + pcTPANS1 exhibited elevated NAb titers (Figure 4D). As expected, mice immunized with the pcTPANS1 DNA vaccine alone showed no NAb, since the NS1 gene encodes a non-structural protein not involved with virus entry.

Taken together, the antibody response analysis directed to the envelope protein shows protective and longstanding seroconversion of animals immunized with the pNS1/E/D2 DNA vaccine and the pE1D2.

### 3.4. Antibody Response against the NS1 Protein Induced by the DNA Vaccines

**Figure 5:**
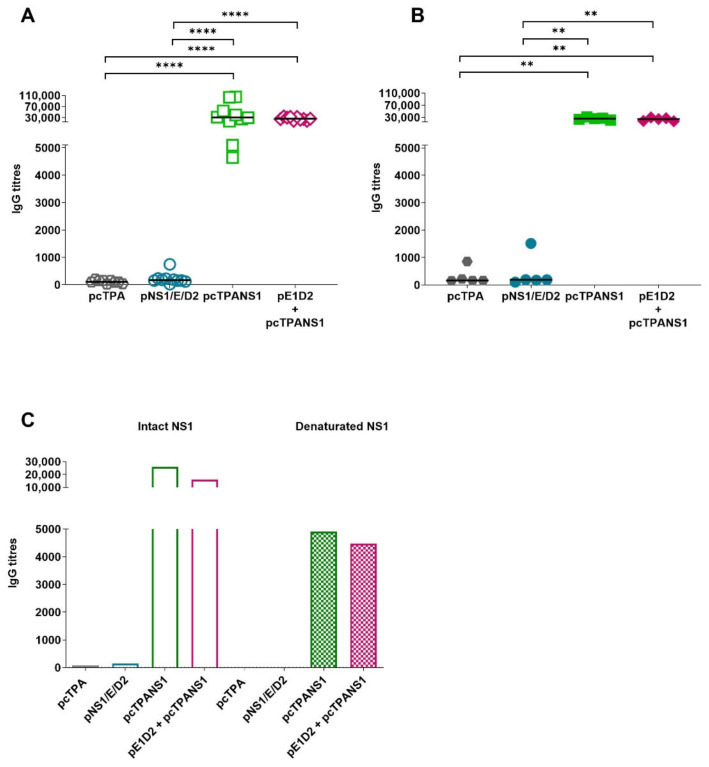
Antibody response against the NS1 protein in serum samples of BALB/c mice immunized with the different DNA vaccines. Antibody titration against the NS1 protein was performed by ELISA using serum samples from mice inoculated with the plasmids pNS1/E/D2, pcTPANS1, pE1D2, or pcTPA alone, as well as with the mixture of pE1D2 + pcTPANS1. Serum samples date from 2 (**A**) or 4 weeks (**B**) after the second DNA dose. Samples from each animal were individually tested in duplicates, and bars represent the median of the obtained values. All ELISA assays were performed using the intact NS1 protein, except in (**C**), in which both intact and heat-denatured proteins were used for testing pooled serum samples of five mice per group (data shown are the median of the obtained values). Asterisks in A and B indicate statistically significant differences between experimental groups using the non-parametric two-tailed Mann–Whitney test (** *p* < 0.01; **** *p* < 0.0001). Data obtained with sera collected 2 weeks after the last immunization are representative of two independent experiments (*n* = 4–5).

Animals were injected with one of the different plasmids or with a mixture of pE1D2 and pcTPANS1. The humoral immune response against the NS1 protein was evaluated by ELISA with BALB/c mouse sera collected two or four weeks after the second DNA dose. The pcTPANS1 vaccine induced high levels of anti-NS1 antibodies when administered alone or in combination with pE1D2 (Figure 5A), and these IgG titers remained high four weeks after the last vaccination (Figure 5B). However, only one pNS1/E/D2 immunized mouse presented a detectable antibody response against the NS1, and no significant anti-NS1 IgG titers were observed in this animal group compared to pcTPA negative control (Figure 5).

ELISA assay performed with denatured NS1 shows an approximately three-fold decrease in anti-NS1 antibody titers of animals vaccinated with pcTPANS1 or pE1D2 + pcTPANS1 compared to IgG levels detected with the intact recombinant protein. Similar to results from the E protein, this decrease indicates that the anti-NS1 antibodies elicited by DNA immunization recognize mainly conformational epitopes of the NS1 protein (Figure 5C).

### 3.5. Activation of the Cellular Immune Response by the DNA Vaccines

The cellular immune response induced by the DNA vaccines was evaluated by secretion of IFN-γ in ELISPOT assays. Splenocytes isolated from immunized mice were stimulated with synthetic peptides contained in the E and NS1 DENV2 proteins.

**Figure 6:**
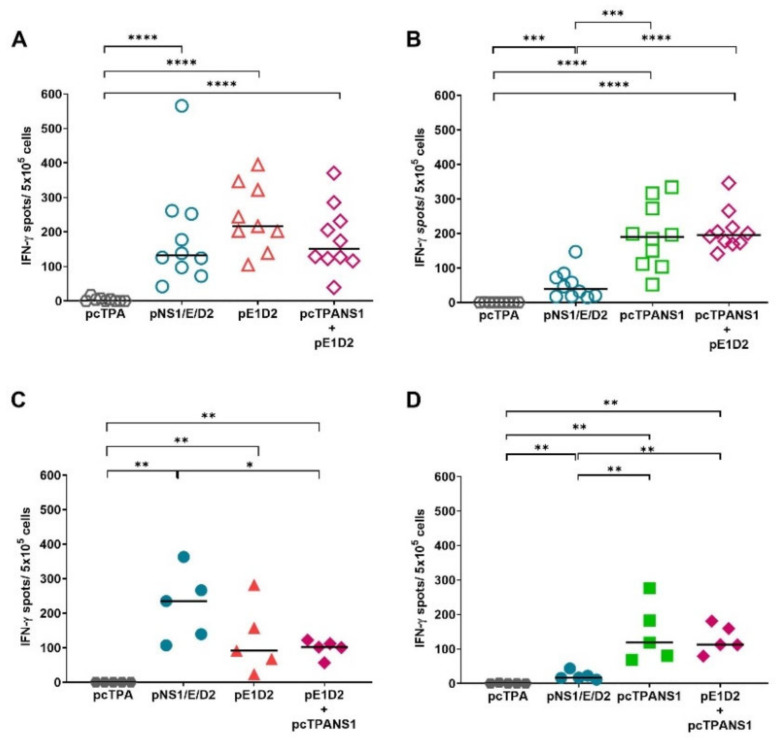
Production of IFN-γ by splenocytes from DNA vaccinated BALB/c mice. Quantification of IFN-γ production by ELISPOT assays using cells from mice immunized with the plasmids pNS1/E/D2, pcTPANS1, pE1D2, and pE1D2 + pcTPANS1, or with the negative control pcTPA (*n* = 5–10). Splenocytes isolated 2 (**A**,**B**) or 4 weeks (**C**,**D**) after the second immunization were incubated in triplicate with (**A**) synthetic peptide *^331^*SPCKIPFEI*^339^* contained in the DENV2 E protein or (**B**) *^265^*AGPWHLGKL*^273^* contained in the DENV2 NS1 protein, both recognized by CD8^+^ T cells; (**C**) pool of 15-mer E-derived peptides or (**D**) pool of 15-mer NS1 derived peptides, with both pools containing peptides that can be recognized by CD4^+^ and CD8^+^ T cells. Values are expressed as the number of spot-forming cells, and bars represent the median of the obtained values. Asterisks indicate statistically significant differences between experimental groups using non-parametric two-tailed Mann-Whitney statistical tests (* *p* < 0.05; ** *p* < 0.01; *** *p* < 0.001; and **** *p* < 0.0001).

Figure 6A,B show IFN-γ production by splenocytes isolated two weeks after the last immunization and incubated with E- and NS1-specific CD8^+^ T cell peptides, respectively. Splenocytes from pNS1/E/D2-vaccinated mice responded to both E- and NS1-derived peptides, as well as the group immunized with the pE1D2 + pcTPANS1 mixture (Figure 6A,B). The number of IFN-γ-producing cells isolated from these two groups was statistically higher when compared to pcTPA-inoculated mice (*p* < 0.0001 for both peptides). The number of cells responsive to the E-derived peptide was statistically equal in the three vaccinated groups: pNS1/E/D2, pE1D2, and pE1D2 + pcTPANS1 (Figure 6A). Regarding the response directed to the NS1 protein, we observed significantly more IFN-γ-producing cells in pcTPANS1- or pE1D2 + pcTPANS1-immunized mice compared to the pNS1/E/D2 group (*p* < 0.001 and *p* < 0.0001, respectively) (Figure 6C).

The specific T-cell response was evaluated 4 weeks after the last vaccination, using pools of E- or NS1-derived peptides screened in a previous work (Figure 6C,D). These peptides were selected from an overlapping 15-mer peptide library and can activate either CD4^+^ or CD8^+^ T cell responses elicited by pE1D2 and pcTPANS1 immunizations [36]. All groups immunized with E-based vaccines responded positively to stimulation with the peptide pool from the E protein. In contrast to stimulation only with the E-derived CD8^+^-specific peptide, IFN-γ production by splenocytes from the pNS1/E/D2 group was statistically higher than the pE1D2 + pcTPANS1 animal group (*p* < 0.01) when stimulated with the peptide pool that can be recognized by CD4^+^ and CD8^+^ T cells. Splenocytes obtained 4 weeks after immunization with pNS1/E/D2, pcTPANS1, and pE1D2 + pcTPANS1 were also positively stimulated with the peptide pool contained in the NS1 (Figure 6D). As expected, cells isolated from pcTPA-inoculated mice did not respond upon stimulation with either E or NS1-derived peptides (Figure 6).

### 3.6. Protection Elicited in BALB/c Mice Immunized with the Different DNA Vaccines

**Figure 7:**
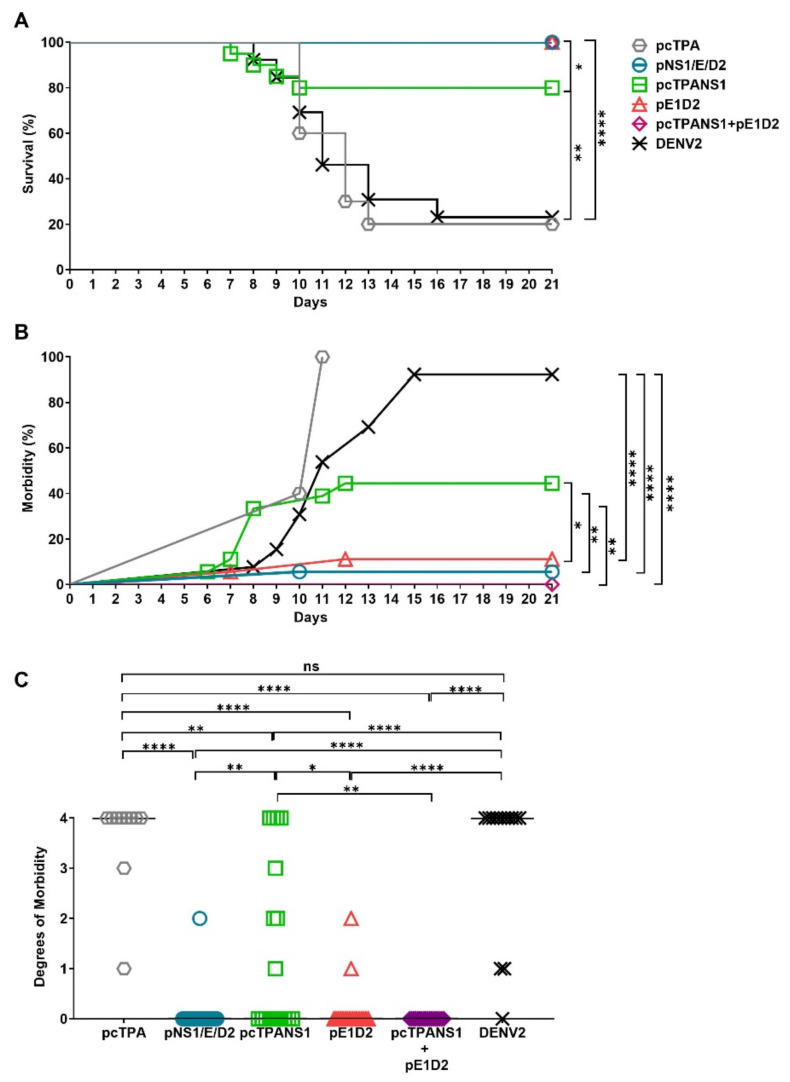
Protection generated in BALB/c mice immunized with the different DNA vaccines after a lethal challenge with DENV2. Animals were i.m. immunized with the different DNA vaccines and monitored for 21 days after DENV2 inoculation by the i.c. route, for assessment of survival (**A**) and morbidity (**B**) rates, and the different morbidity degrees (**C**). A semi-quantitative analysis of the clinical signs of infection was made using a morbidity degree scale from 0 to 4: 0 = absence of clinical signs; 1 = paralysis in one hind leg or alteration of the spinal column; 2 = severe paralysis in one leg and alterations of the spinal column or severe paralysis on both hind legs; 3 = severe paralysis in the hind legs and alterations of the spinal column; and 4 = death. Lines represent the median of the observed degrees (**C**). Asterisks indicate significant differences between experimental groups using Log-Rank (Mantel–Cox) test in (**A**,**B**) or the non-parametric two-tailed Mann–Whitney statistical test in (**C**) (* *p* < 0.05; ** *p* < 0.01; and **** *p* < 0.0001), ns: non-significant. Data are representative of two independent experiments (*n* = 8–10). Significant differences between pcTPA and other experimental groups in (A, B) are the same as for the non-immunized group.

The protection conferred by the different DNA vaccines was evaluated in immunized BALB/c mice, followed by a lethal DENV2 challenge. Animals were monitored 21 days after the viral challenge with daily morbidity and mortality records. Immunization with the vaccines pNS1/E/D2, pE1D2, and the mixture pE1D2 + pcTPANS1 resulted in a 100% survival rate against the DENV2 (Figure 7A). The pcTPANS1 vaccine led to 80% survival after the dengue infection. On the other hand, only 23% of non-immunized animals survived after the DENV2 inoculation by the intracerebral route, thus confirming the lethality of the virus inoculum employed for the challenge. All immunized groups showed statistically different survival rates compared to the non-immunized group or animals inoculated with the pcTPA control plasmid (Figure 7A). Clinical signs began from the 7th to the 10th day post-infection (dpi) and ended on the 16th dpi in the non-immunized mice, with a 92% morbidity rate (Figure 7B). In contrast, none of the pE1D2 + pcTPANS1-immunized animals showed any clinical sign of infection. We observed 5% and 10% morbidity rates in pNS1/E/D2- and pE1D2-immunized mouse groups, respectively, with the clinical infection signs appearing until 12 dpi. On the other hand, almost half of the pcTPANS1-inoculated animals (44%) were affected by the DENV2 challenge (Figure 7B).

The clinical signs of infection were classified on an arbitrary morbidity scale, ranging from 0 to 4 degrees, concerning mainly leg paralysis and commitment of the spinal column with different severity degrees (Figure 7C). The group of pcTPANS1-immunized animals showed varying degrees of morbidity but was statistically different from the pcTPA-inoculated or non-immunized control groups. On the other hand, the morbidity observed in pNS1/E/D2- and pE1D2-immunized animal groups did not exceed degree 2. Furthermore, no morbidity was detected in animals immunized with the mixture pE1D2 + pcTPANS1. These results reinforce the protection generated after immunization with DNA vaccines mediating the simultaneous expression of the NS1 and E proteins.

## 4. Discussion

After decades of effort, dengue persists as an impacting endemic disease that urgently requires the development of a protective and safe vaccine [43]. The only licensed vaccine, Dengvaxia, consists of four live-attenuated chimeric viruses with the backbone of yellow fever virus and prM/E from the four dengue serotypes. However, this vaccine is not recommended for DENV seronegative individuals, mainly children, since they become more susceptible to developing severe dengue when infected after vaccination, probably by the effect of antibody-dependent enhancement (ADE) of virus replication. These results suggest that other DENV antigens not present in Dengvaxia, such as the non-structural proteins, may be necessary to generate robust protection by inducing critical T cell responses [7,8,9]. Two other vaccines in clinical trials are based on one or more attenuated dengue viruses and the replacement of prM/E genes of different serotypes [44].

In the present work, we bet on combining the DENV2 E and NS1 genes either in a bicistronic DNA vaccine or by using two plasmids encoding these proteins. We have previously constructed the DNA vaccines pE1D2 and pcTPANS1 that encode the ectodomains of the DENV2 E protein and the NS1, respectively [29,33]. Both vaccines were extensively studied and have been shown to induce protective humoral and cellular immune responses in BALB/c mice. Therefore, in the present work, we evaluated the combination of these two vaccines (pE1D2 + pcTPANS1) and the new construct pNS1/E/D2, which encodes both proteins E and NS1 concomitantly.

All our DNA vaccines contain the sequence encoding the t-PA signal peptide to direct the recombinant proteins to the secretory pathway. This peptide is well known and efficiently used in nucleic acid-based vaccines. In the case of plasmids encoding the E ectodomain, our results suggest that the absence of the prM/M protein, which acts as a chaperonin during dengue infection, seems not to impact in the secretion of the recombinant E protein. Moreover, our ELISA results with intact and heat-denatured antigen suggest that the E ectodomain folding is similar to that of the native protein and, therefore, induced mainly antibodies against conformational epitopes.

Our results showed that immunization with the pNS1/E/D2 triggered broad immune responses, including neutralizing antibodies and T cell activation with the production of IFN-γ, and led to protection in BALB/c mice challenged with DENV2. These findings were also valid for mice vaccinated with the combination of the pE1D2 and pcTPANS1 plasmids. Interestingly, the combination of pE1D2 + pcTPANS1 DNA vaccines provided complete protection without any clinical sign of infection after the DENV2 challenge, which was not observed after immunization with each of these vaccines isolatedly. The results suggest that the induction of immune responses against these two proteins may be more effective by eliciting NAb and preventing, in later stages, the increase of infection derived from a virus that escapes neutralization.

During the dengue infection, the NS1 plays a role in the pathogenesis. Reports suggest its involvement in hepatic damage or the enhancement of the virus infection. However, our previous work with the pcTPANS1 vaccine revealed no hepatotoxicity after the in vivo expression of the NS1 in vaccinated mice, without the histopathological effects or serum level increase of the hepatic enzymes [32]. Moreover, immunization with either the pcTPANS1 or the pNS1/E/D2 generated protection after the virus challenge, thus indicating no enhancement of the dengue infection.

Other studies also reported the antigenicity of the E and NS1 proteins of the dengue virus using different vaccine platforms [45]. Most of these studies are based on cloning the sequences encoding the E and NS1 proteins fused into a single fragment so that a single hybrid protein is expressed [46,47,48,49]. However, the correct folding of the resulting hybrid protein is a major concern, and the conservation of specific conformational antigenic domains may be closely linked to the immunogenic function of these proteins, especially for the induction of protective antibodies. One advantage of DNA vaccines, in turn, is the endogenous expression of the antigen, which conserves its conformational native epitopes and stimulates the humoral and cellular immune responses. There are two DNA dengue vaccines based on prM/E that are in clinical trials [43]. Notably, the first DNA vaccine for human use was approved recently against SARS-CoV-2, the virus causing the massive pandemic of COVID-19 [50].

The strategy addressed to construct pNS1/E/D2 consisted of a bicistronic plasmid DNA vaccine harbouring the DENV2 E and NS1 genes under the control of CMV and SV40 promoter regions, respectively. Ideally, a single vector containing two separate transcription units could promote synergistic responses with the two viral antigens expressed simultaneously [51]. Immunofluorescence analysis of BHK-21 cells transfected with the pNS1/E/D2 DNA vaccine showed simultaneous expression of the E and NS1 proteins, thus confirming the ability of this plasmid to mediate the simultaneous production of these antigens. Moreover, the mass spectrometric analysis of the supernatant of transfected cells confirmed the secretion of both proteins. However, the flow cytometry and mass spectrometry results showed that the expression and secretion of the NS1 protein driven by the newly constructed pNS1/E/D2 vaccine were lower when compared to expression mediated by the pcTPANS1.

In contrast, the E protein was equally expressed in cells transfected with any recombinant DNA vaccines. The expression of the E protein may negatively interfere with the efficiency of simultaneous NS1 expression. Another explanation may be related to the efficiency of the CMV and SV40 promotor regions used in the pNS1/E/D2. The great majority of DNA vaccines use the CMV region to control the transcription of various genes [51]. We first attempted to construct a DNA vaccine that would encode the two dengue genes under the control of the CMV promoter in independent expression cassettes. Unfortunately, this strategy proved unfeasible, probably due to the repetition of a large region in the same plasmid. On the other hand, when we immunized animals with the combination of the pcTPANS1 and pE1D2 vaccines, the expression of both proteins was controlled by the CMV promoter.

The immune response induced in immunocompetent BALB/c mice inoculated with the pNS1/E/D2, and the combination of the pE1D2 + pcTPANS1 vaccines was then investigated and compared to the response observed in animals immunized with each plasmid individually. Results based on ELISA and PRNT assays showed that immunization with pNS1/E/D2 elicited a specific anti-E antibody response similar to pE1D2, including the production of antibodies with neutralizing activity against DENV2. Numerous groups have already demonstrated the impact of NAb in the protection afforded by several vaccine prototypes based on the E protein of DENV [43]. Moreover, Lu and colleagues showed an increase in NAb titers induced in mice immunized with a DNA vaccine containing the prM/E/NS1 genes from DENV2 compared to the formulation lacking the NS1 protein [49]. In our study, all three tested E-based DNA vaccine formulations triggered similar anti-E IgG and NAb levels, showing the efficacy of the pNS1/E/D2 plasmid in inducing an effective humoral immune response against the E protein. Additionally, this result indicates that the presence of NS1 did not affect the magnitude of this response.

Conversely, several shreds of evidence support the benefits of using the NS1 in a dengue vaccine by inducing a protective antibody response. Immunization with NS1-based vaccines, the injection of recombinant NS1 protein, and the passive immunization with anti-NS1 antibodies have shown to protect mice against DENV and other related viruses such as the Yellow Fever virus, West Nile virus, and Zika virus [25,27,29,30,32,34,52,53,54,55,56]. In our study, we observed high levels of anti-NS1 IgG in animals immunized with pcTPANS1 or the mixture pE1D2 + pcTPANS1 but not in pNS1/E/D2-vaccinated mice. Such results may be directly related to the lower NS1 expression mediated by the pNS1/E/D2, as demonstrated by the in vitro experiments with transfected cells, which compromised the induction of anti-NS1 antibodies.

Concerning the specificity of anti-E and anti-NS1 antibodies generated by our DNA vaccines, we observed a dramatic decrease in antibody titers when the heat-denatured recombinant proteins were used in the ELISA assays, revealing that most of the elicited antibodies recognized the conformational epitopes of the E and NS1 proteins. These are promising results since murine and human reports on dengue immunoglobulin responses have shown a protective role of antibodies directed to conformational epitopes present in such proteins [57,58,59].

Besides the involvement of antibody responses, the importance of the cellular immune response in protection against dengue has been increasingly pointed out [20,31,60,61,62,63,64,65]. It also highlights the potential of incorporating non-structural proteins in vaccine candidates against dengue since they are the major targets of cellular immunity. We have previously identified NS1- and E-derived epitopes recognized by both CD4^+^ and CD8^+^ T cells from mice immunized with pcTPANS1 or pE1D2 vaccines [36]. Hence, we used these peptides in our ELISPOT assays conducted 4 weeks after the last immunization and observed that they were also recognized by splenocytes isolated from pNS1/E/D2-vaccinated mice.

In the present work, we showed that the pNS1/E/D2 DNA vaccine was able to activate a T-cell response with IFN-γ production upon stimulation with specific CD4^+^ and CD8^+^ synthetic peptides derived from either the E or the NS1 proteins. Data also revealed that all mice immunized with the DNA vaccines encoding E and NS1 proteins alone or in combination exhibited IFN-γ producing cells, emphasizing the differential ability of DNA vaccines to induce a robust cellular immune response. Although the expression of NS1 protein mediated by the pNS1/E/D2 seemed less efficient, with no induction of significant anti-NS1 antibodies, mice immunized with this DNA vaccine did present an NS1-specific cellular response showing that, even with low recombinant protein expression, a T cell activation may occur.

To investigate the protection elicited by the DNA vaccines, animals were challenged intracerebrally with a neuroadapted DENV2. None of the available murine models for studying dengue are able to mimic all the aspects of the dengue disease observed in humans. One possibility is the use of immunocompromise mice, in particular those deficient in the IFN-α/β and IFN-γ pathways, which develop some clinical signs after infection with DENV similar to those observed in humans [66]. However, these animals present altered innate and adaptive responses that have a strong impact on dengue infection, and, consequently, their use for vaccine tests is controversial [67]. Immunocompetent mice, in turn, may be important due to their integrity of the immune system. In fact, dengue infection by the intracerebral route is the classical test for dengue vaccines, although it is focused on the virus neurotropism and does not reproduce all the dengue symptoms [29,33,68,69,70]. One of the limitations of this infection model is the decrease in the animal’s susceptibility to the viral challenge as they get older. Moreover, older mice have a thicker skull, which makes the intracerebral injection of DENV2 more difficult in animals older than 8 weeks of age. However, our previous results [34] and the data in the present work support long-term protection elicited by the DNA vaccines.

Other studies also reported protection by DNA vaccines based on the DENV E and NS1 proteins but in lower magnitudes. Lu and collaborators [49] showed partial protection (40% survival) conferred by a DNA vaccine encoding DENV2 prM/E/NS1 with immunization and challenge models similar to those used in the present work. Another report, with a DNA vaccine based on the NS1 gene and the sequence encoding the domains II/III of the E protein from DENV2, showed the induction of anti-NS1 antibodies and anti-E NAb in mice after a prime boost strategy with recombinant proteins, although in vivo protection tests were not performed [47].

These data, taken together with our results, reinforced that the combined and complementary immunogenicity of antibodies and T-cells specific for the E and NS1 proteins of DENV contribute to robust protection. Further studies regarding bicistronic expression technology may be fundamental to improving the immunogenicity of the pNS1/E/D2 DNA vaccine, mainly concerning the better efficacy of the NS1 protein expression.

## Figures and Tables

**Table 1:** Mass spectrometric identification of dengue virus proteins in the supernatant of BHK-21 cells transfected with different DNA vaccines.

Gel Fraction ^(a)^	DNA Vaccine ^(b)^	Protein ^(c)^	Mass ^(d)^	UniquePep ^(e)^	UniqueSC ^(f)^	NSAF ^(g)^	%Coverage ^(h)^	PtnScore ^(i)^
F3-F4	pcTPANS1	NS1	42,525	87	627	0.2637	84	206.65
F7-F8	pE1D2	E	46,881	31	85	0.0379	67	82.73
F9-F10	pE1D2 + pcTPANS1	NS1	42,525	66	484	0.1838	80	168.49
		E	46,881	31	71	0.0239	65	80.26
F11-F12	pNS1/E/D2	NS1	42,525	25	58	0.0293	55	53.17
		E	46,881	30	62	0.0278	61	80.38

^(a)^ SDS-PAGE fraction used for in-gel digestion and protein identification by nLC-MS/MS; ^(b)^ plasmid DNA used to transfect BHK-21 cells; ^(c)^ name of the identified protein; ^(d)^ theoretical molecular mass of the protein in kDa; ^(e)^ number of identified peptides exclusive to the protein sequence; ^(f)^ total number of spectral counts of all identified unique peptides mapping to the protein sequence; ^(g)^ relative protein abundance based on the “Normalized Spectral Abundance Factor”; ^(h)^ percentage of identified sequence residues of the protein; and ^(i)^ protein confidence identification score calculated by the algorithm PatternLab V.

## Supplementary Materials

The following supporting information can be downloaded at: https://www.mdpi.com/article/10.3390/v14071452/s1, Figure S1: Schematic representation of the NS1 cassette cloning strategy into the plasmid pE1D2; Figure S2: Flow cytometry analyses of BHK-21 cells transfected with the DNA vaccines to quantify expression of E and NS1 proteins; Figure S3: SDS-PAGE of the supernatant of BHK-21 cells transfected with different DNA vaccines; Figure S4: Detailed information on dengue virus proteins identified by MS/MS in the supernatant of BHK-21 cells transfected with different DNA vaccines; Table S1: List of all proteins identified by MS/MS in the supernatant of BHK-21 cells transfected with different DNA vaccines.
