# Supplementary material for: Combination of E- and NS1-Derived DNA Vaccines: The Immune Response and Protection Elicited in Mice against DENV2
Source: Viruses. 2022 Jun 30;14(7):1452. doi: 10.3390/v14071452 (PMC9323404; doi:10.3390/v14071452)
Supplement: Supplementary file 1 [file viruses-14-01452-s001.zip › Figure S1-S3.pdf]

Supplemenary Material

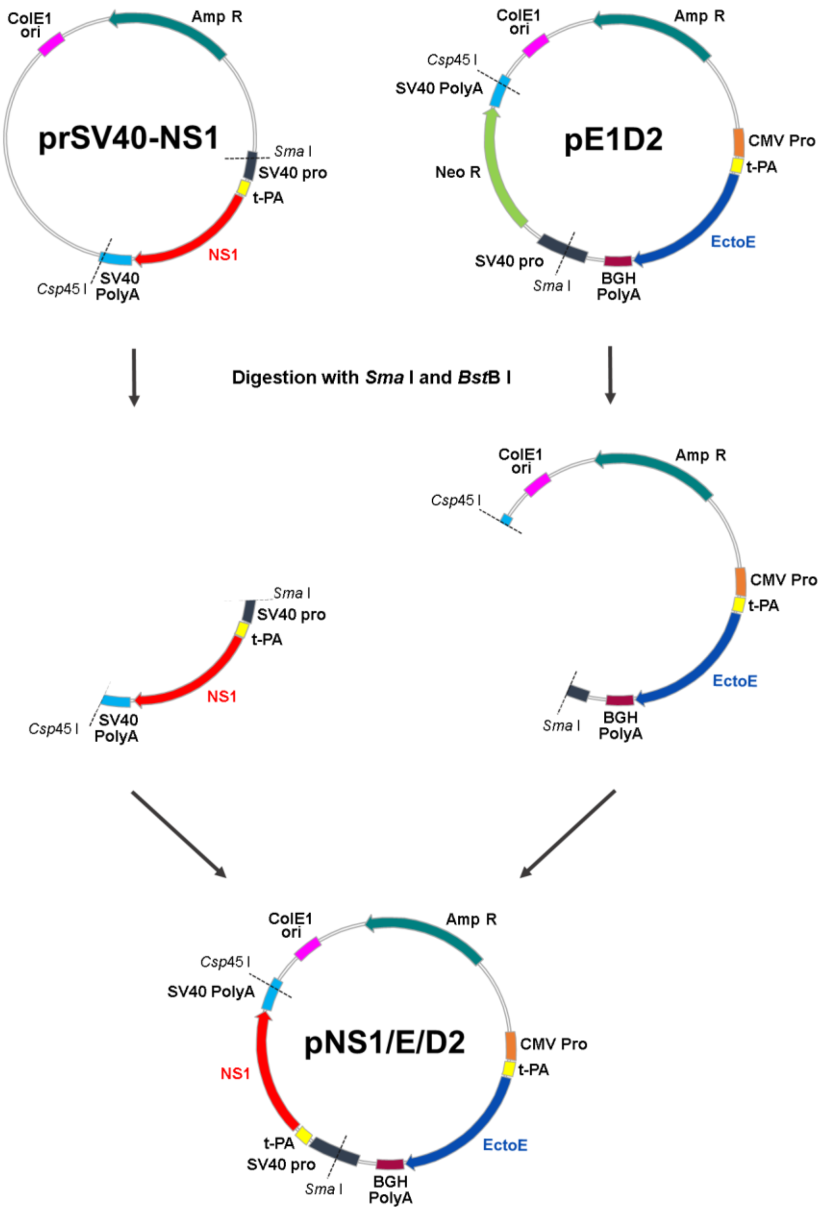

Figure S1: Schematic representation of the NS1 cassette cloning strategy into the plasmid pE1D2. The prSV40-NS1 plasmid containing the NS1 cassette and the pE1D2 plasmid were restricted with *Sma* I and *Csp*45 I enzymes, and the resulting fragments were ligated. Amp R - ampicillin resistance gene; CMV pro - cytomegalovirus promoter; EctoE - sequence coding the ectodomain region of the E protein; BGH polyA - bovine growth hormone polyadenylation signal; SV40 pro - Simian virus 40 promoter; NS1 - sequence coding the NS1 protein; SV40 polyA - Simian virus 40 polyadenylation signal; ColE1 ori - *Escherichia coli* replication origin.

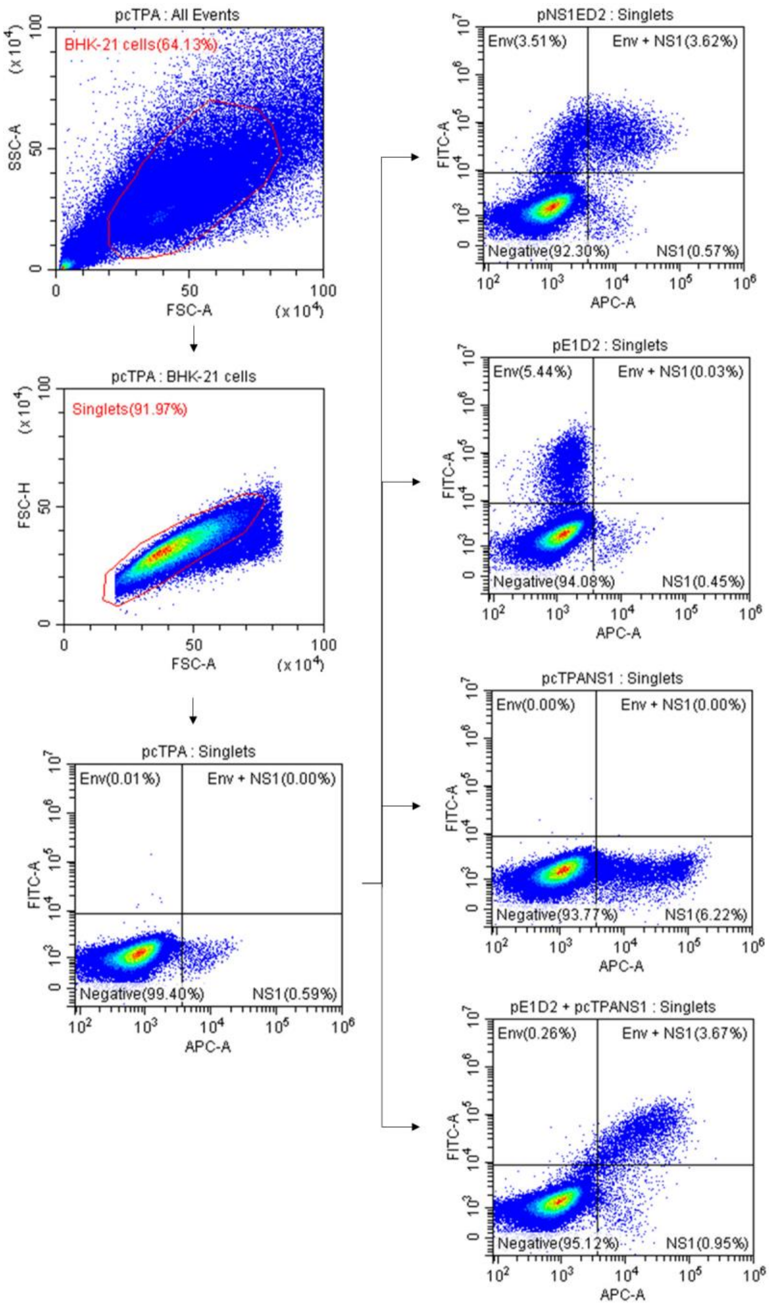

Figure S2: **Flow cytometry analyses of BHK-21 cells transfected with the DNA vaccines to quantify expression of E and NS1 proteins.** Representative flow cytometry dot plots displaying E- and NS1-positive frequencies in BHK-21 cells transfected with the control pcTPA or with the DNA vaccines pNS1/E/D2, pE1D2, pcTPANS1, and pE1D2 + pcTPANS1. Transient transfection was carried out with Lipofectamine reagent. After the 24h-transfection, cells were harvested and stained with specific antibodies for E and NS1 detection: mouse monoclonal anti-DENV2 3H5 antibody and rabbit polyclonal anti-NS1 antibodies, followed by incubation with goat anti-rabbit IgG conjugated to Alexa Fluor 488, and goat anti-mouse IgG conjugated to Alexa Fluor 546. Gates for selecting populations containing single BHK-21 cells (A, B). Negative and positive staining for E and NS1 proteins were gated in BHK-21 cells transfected with the negative control pcTPA (C). Each subsequent dot plot shows E- or/and NS1- positive populations after transfection with each DNA vaccine based on pcTPA-transfected cells gates. The values of unspecific staining of E and NS1 proteins in pcTPA-

transfected cells were subtracted from pNS1/E/D2, pE1D2, pcTPANS1, and pE1D2 + pcTPANS1 groups.

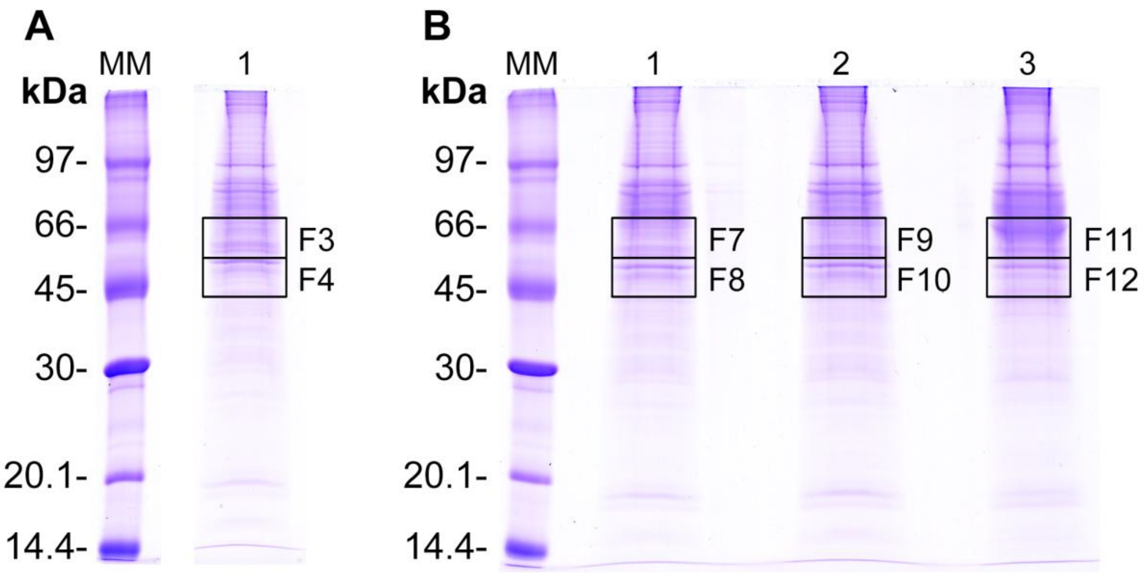

Figure S3: SDS-PAGE of the supernatant of BHK-21 cells transfected with different DNA vaccines. A) MM: molecular mass standards; 1: Transfection with pcTPANS1. B) MM: molecular mass standards; 1) Transfection with pE1D2; 2) Transfection with pE1D2 + pcTPANS1; 3) Transfection with pNS1/E/D2. The fractions used for in-gel digestion and MS/MS identification are indicated with black rectangles. All samples were run under reducing conditions and the gels (12 %T) were stained with Coomassie blue R250.
