# Supplementary material for: Combination of E- and NS1-Derived DNA Vaccines: The Immune Response and Protection Elicited in Mice against DENV2
Source: Viruses. 2022 Jun 30;14(7):1452. doi: 10.3390/v14071452 (PMC9323404; doi:10.3390/v14071452)

pcTPANS1 (fractionsF3&F4): Dengue virus proteins identified (with protein sequence coverage)

PatternLab for proteomics :: SEPro :: F3&F4.sepr2

Load Identify Project Organization Select Utils Help

Save Statistics Tools

Spec FDR: 0 / 2134 = 0% Pep FDR: 0 / 524 = 0% Prot FDR: 0 / 45 = 0% # Prot (Max Parsimony): 0 / 33 = 0% Unique Prot: 30 Unlabeled Decoys: 0 / 0

View mode: (•) Proteins ( ) Proteins Max Pars ( ) Peptides ( ) Scans [ ] Inferred Protein Families DENV2

	Locus	Length	#UniquePeptides	MolWt	SequenceCount	SpectrumCount	SpectrumCountUnique	NSAF	Coverage	Protein Score	Description
1	TPA_NS1_377aa_DENV2	377	87	42524.9	87	627	627	0.2637055	0.8382	206.646	

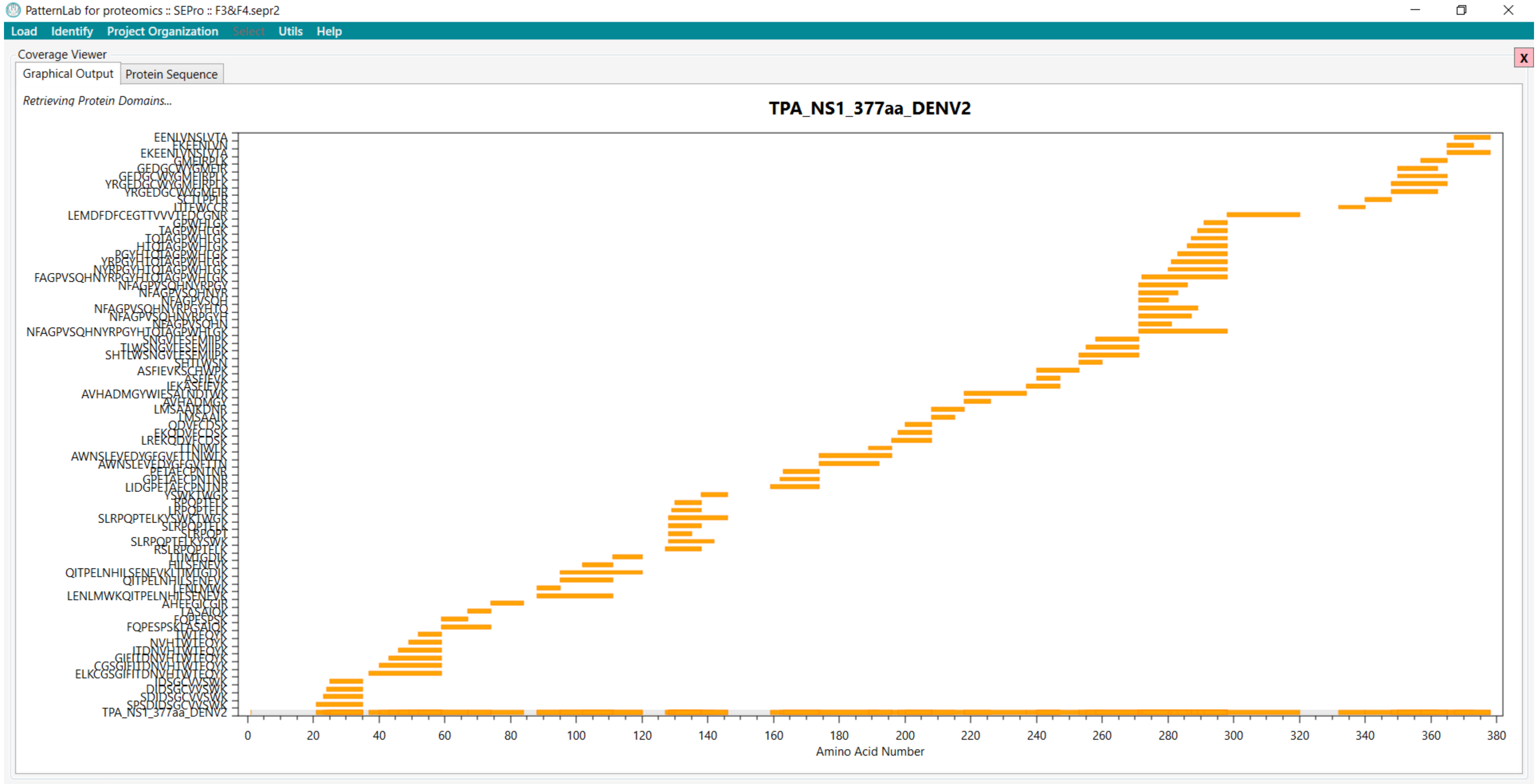

pcTPANS1 (fractionsF3&F4): Best MS/MS spectrum identifying protein “TPA_NS1_377aa_DENV2” (top 1 primary score)

	File Name	Scan No	Z	Unique	MZ	MeasuredMH	TheoreticalMH	PPM	PrimaryScore	SecondaryScore	DeltaCN	PeaksMatched	Ret Time	Classification Score	LeftAA	PeptideSequence	RightAA
1	20211003_Ada2_pcTPA_NS1_F3	27016	2	True	1029.0234	2057.039598	2057.037188	1.1716	5.3038	40.179584	0.8244	24	62.53	0.8925637349493519	K	SHTLWSNGVLESEM[15.9949]IIPK	N

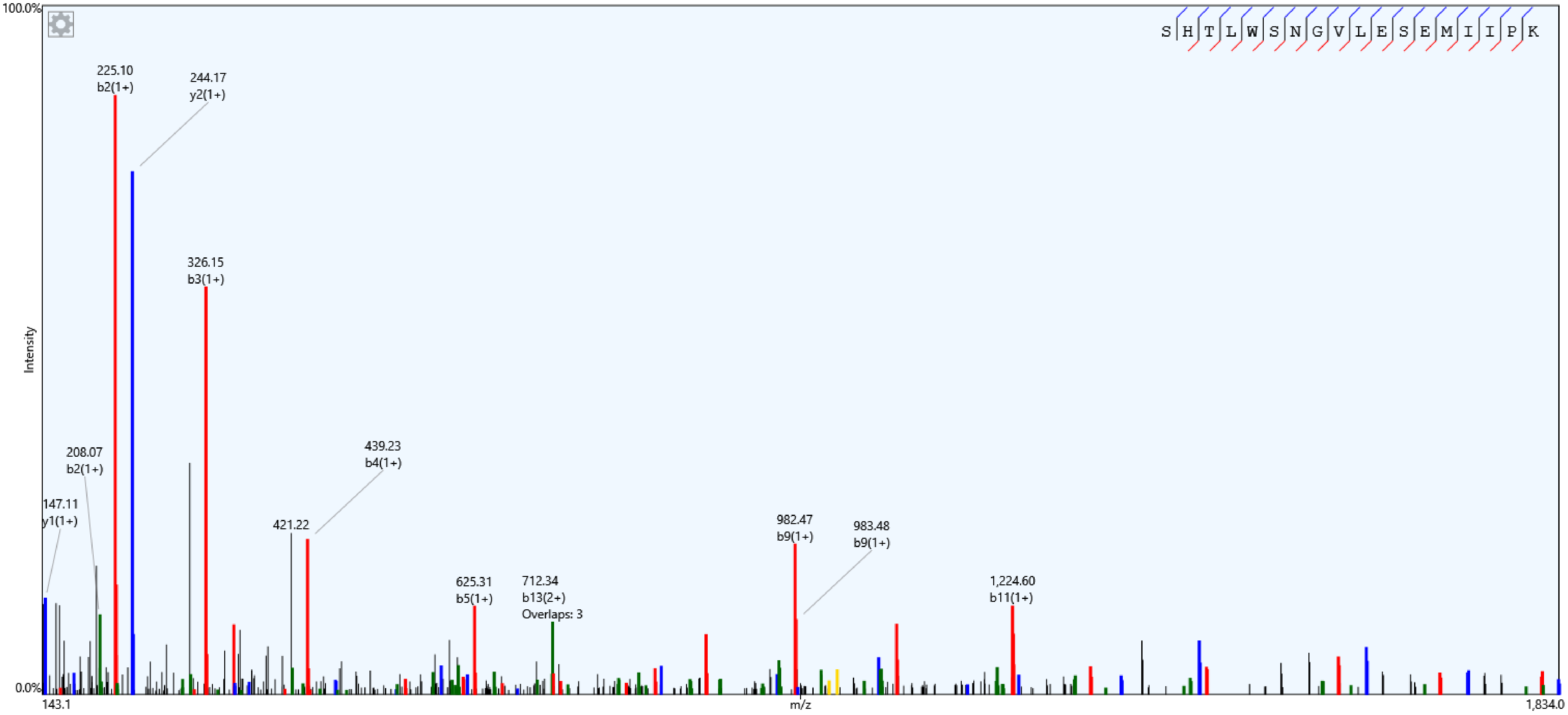

pE1D2 (fractionsF7&F8): Dengue virus proteins identified (with protein sequence coverage)

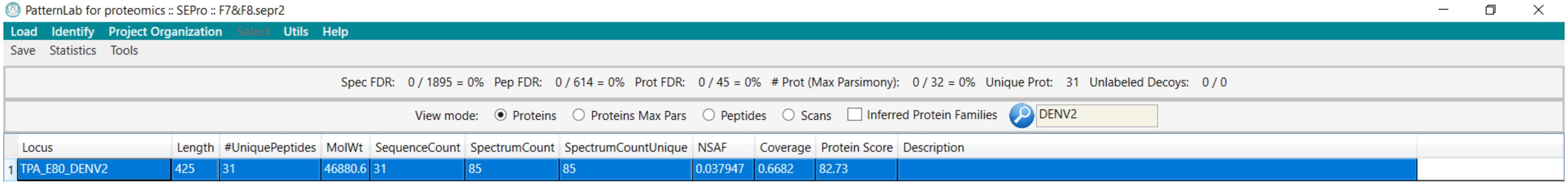

	Locus	Length	#UniquePeptides	MolWt	SequenceCount	SpectrumCount	SpectrumCountUnique	NSAF	Coverage	Protein Score	Description
1	TPA_E80_DENV2	425	31	46880.6	31	85	85	0.037947	0.6682	82.73	

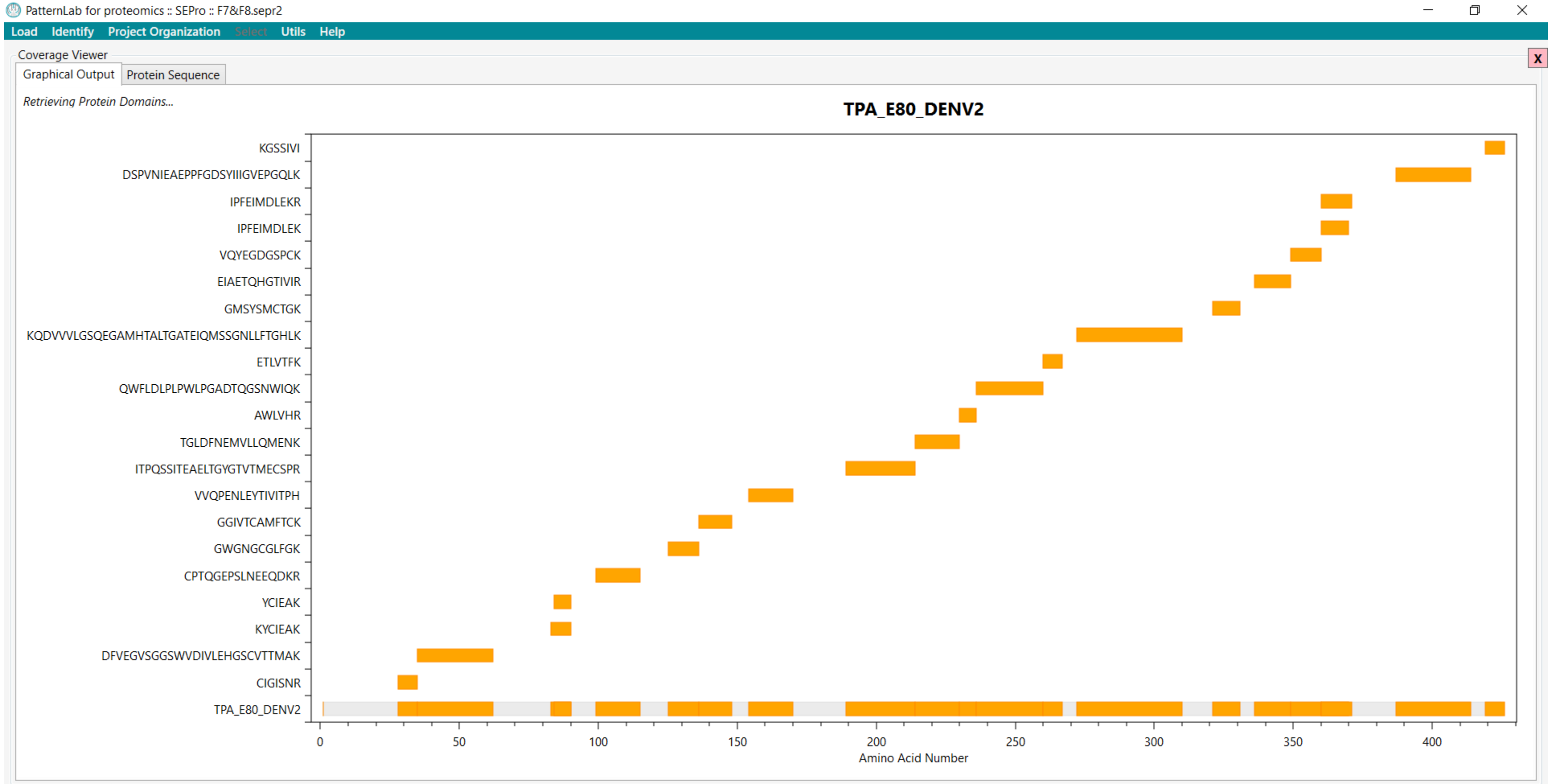

pE1D2 (fractionsF7&F8): Best MS/MS spectrum identifying protein "TPA_E80_DENV2" (top 1 primary score)

	File Name	Scan No	Z	Unique	MZ	MeasuredMH	TheoreticalMH	PPM	PrimaryScore	SecondaryScore	DeltaCN	PeaksMatched	Ret Time	Classification Score	LeftAA	PeptideSequence	RightAA
1	20211003_Ada4_pE1_D2_2020_F7	34380	3	True	960.7916	2880.360247	2880.354253	2.081	5.1959	27.5918	0.8014	27	74.57	0.6957180772962759	R	DFVEGVSGGSWVDIVLEHGSCVTTMAK	N

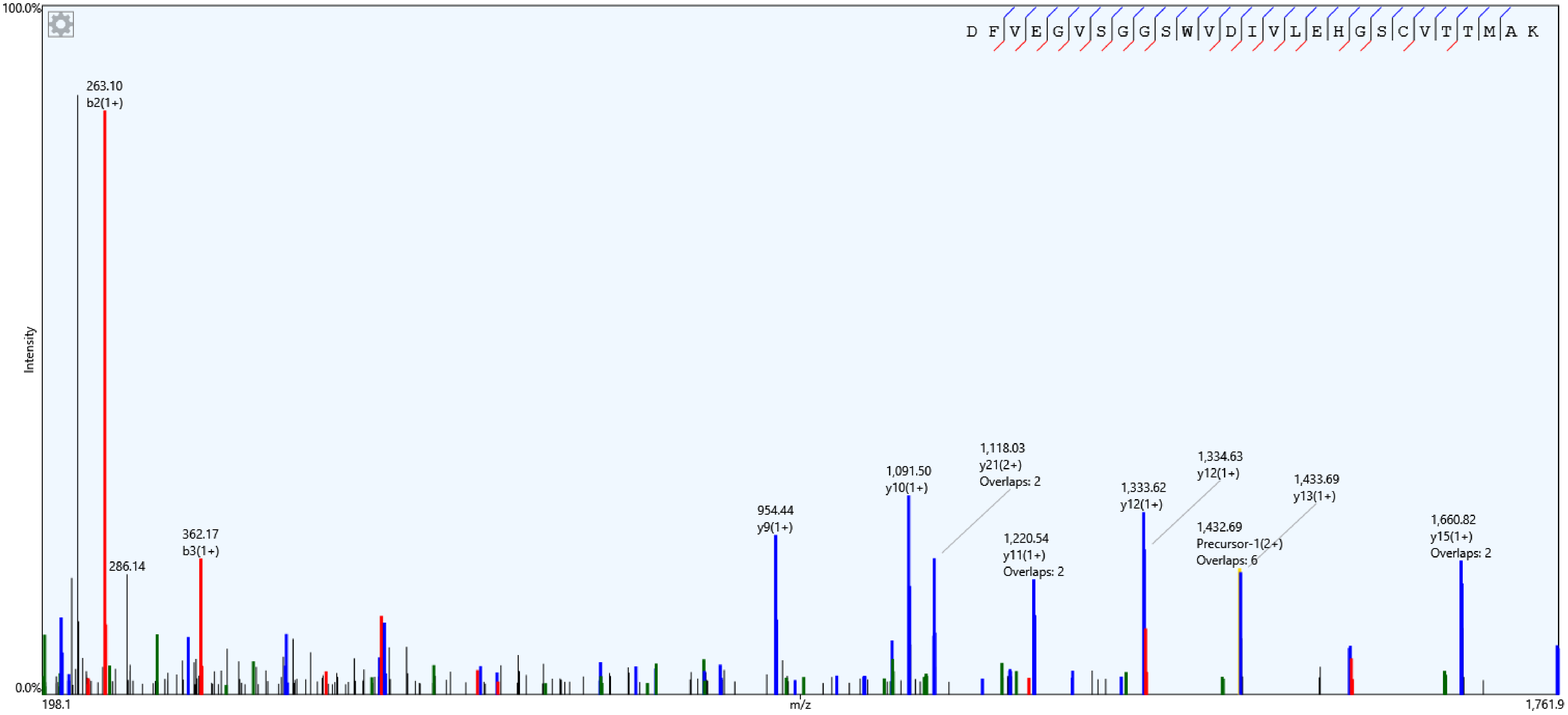

pE1D2 + pcTPANS1 (fractionsF9&F10): Dengue virus proteins identified (with protein sequence coverage)

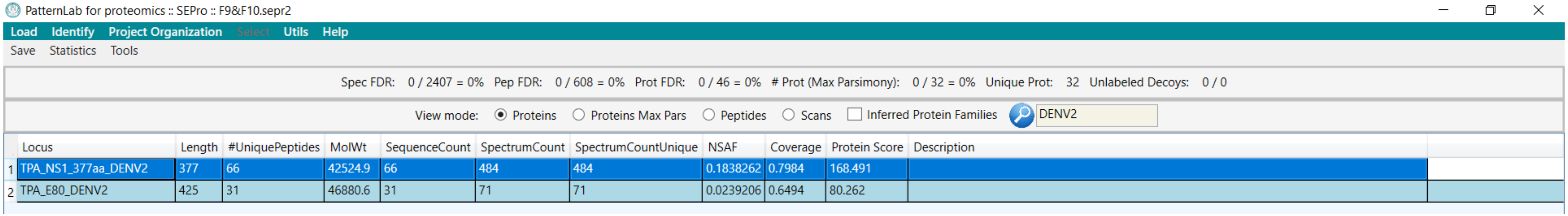

	Locus	Length	#UniquePeptides	MolWt	SequenceCount	SpectrumCount	SpectrumCountUnique	NSAF	Coverage	Protein Score	Description
1	TPA_NS1_377aa_DENV2	377	66	42524.9	66	484	484	0.1838262	0.7984	168.491	
2	TPA_E80_DENV2	425	31	46880.6	31	71	71	0.0239206	0.6494	80.262	

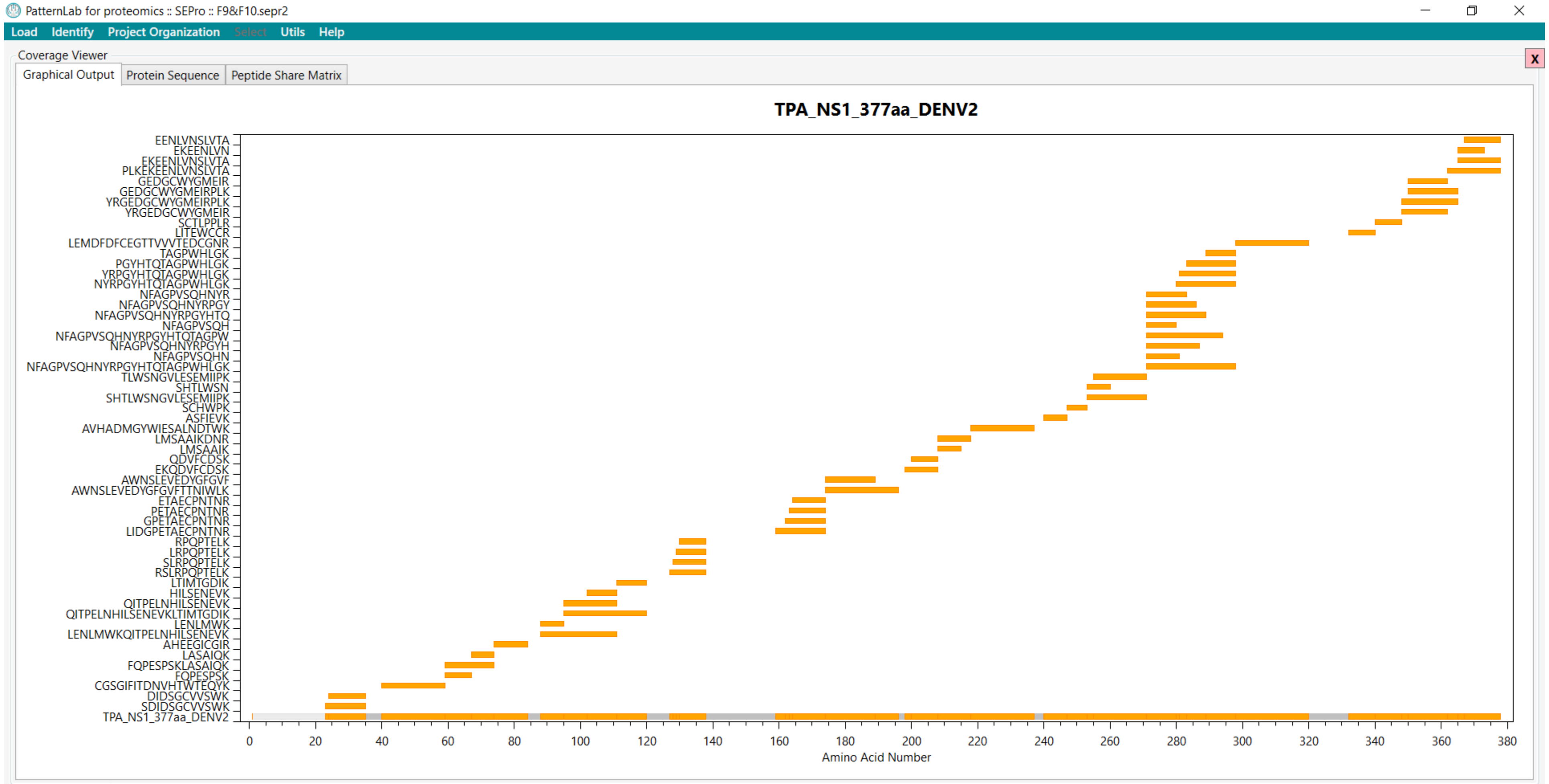

pE1D2 + pcTPANS1 (fractionsF9&F10): Best MS/MS spectrum identifying protein “TPA_NS1_377aa_DENV2” (top 1 primary score)

	File Name	Scan No	Z	Unique	MZ	MeasuredMH	TheoreticalMH	PPM	PrimaryScore	SecondaryScore	DeltaCN	PeaksMatched	Ret Time	Classification Score	LeftAA	PeptideSequence
1	20211003_Ada5_pE1_D2_2020_pcTPA_NS1_F9	29687	2	True	1021.0273	2041.047411	2041.042288	2.51	5.2262	31.220961	0.7962	25	65.36	0.7830536601120683	K	SHTLWSNGVLESEMIIPK

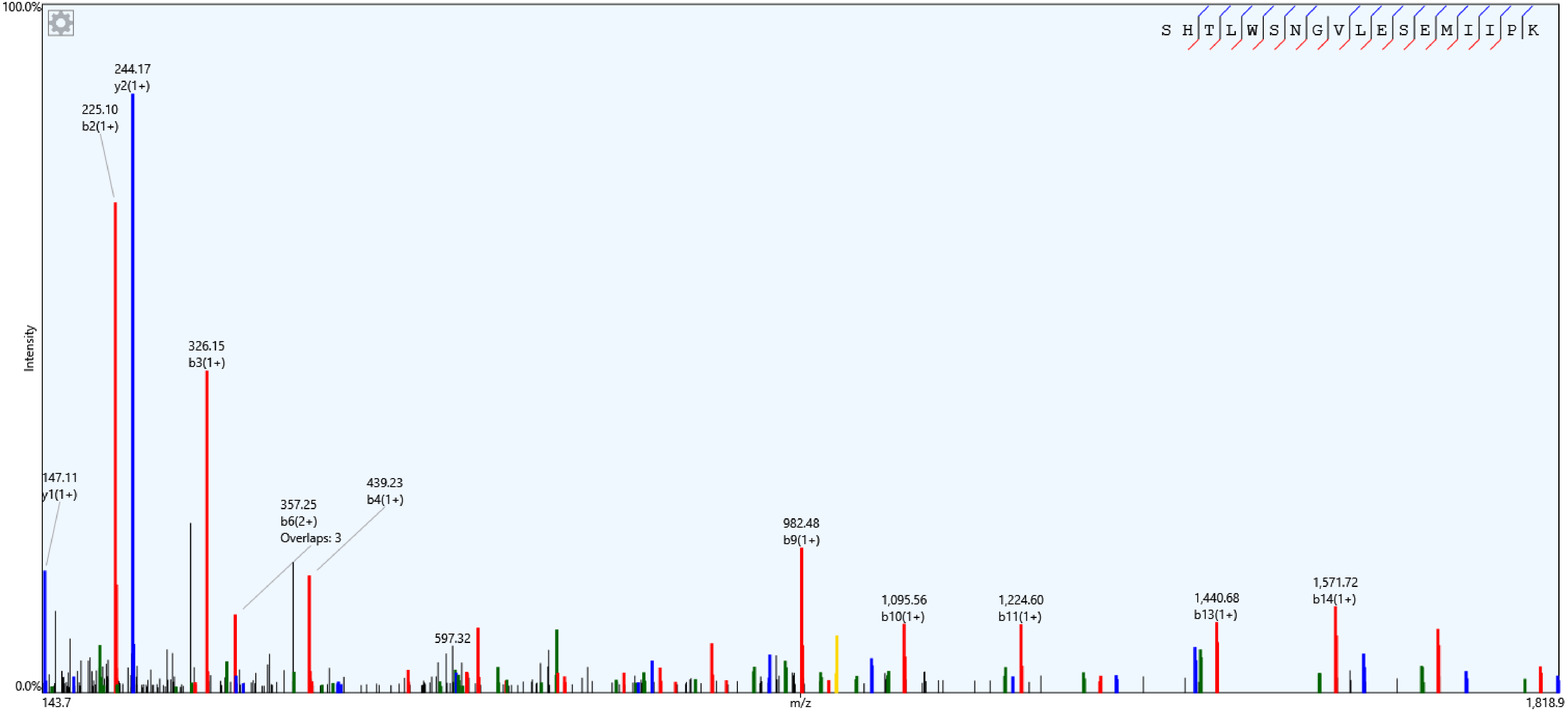

pE1D2 + pcTPANS1 (fractionsF9&F10): Dengue virus proteins identified (with protein sequence coverage)

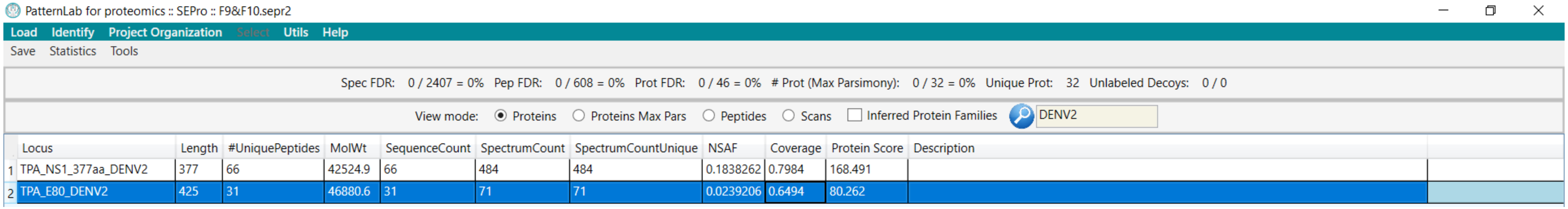

	Locus	Length	#UniquePeptides	MolWt	SequenceCount	SpectrumCount	SpectrumCountUnique	NSAF	Coverage	Protein Score	Description
1	TPA_NS1_377aa_DENV2	377	66	42524.9	66	484	484	0.1838262	0.7984	168.491	
2	TPA_E80_DENV2	425	31	46880.6	31	71	71	0.0239206	0.6494	80.262	

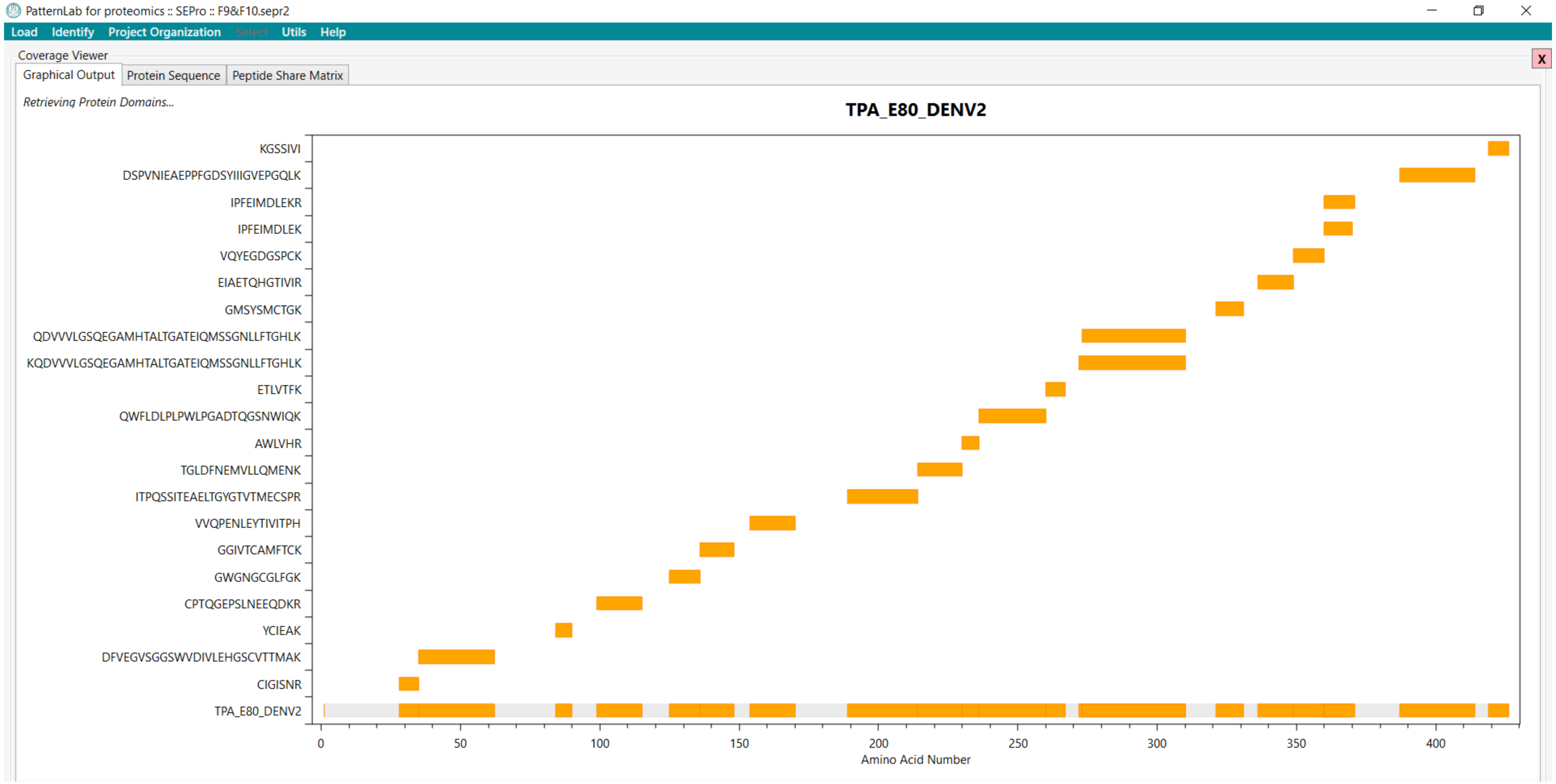

pE1D2 + pcTPANS1 (fractionsF9&F10): Best MS/MS spectrum identifying protein "TPA_E80_DENV2" (top 1 primary score)

	File Name	Scan No	Z	Unique	MZ	MeasuredMH	TheoreticalMH	PPM	PrimaryScore	SecondaryScore	DeltaCN	PeaksMatched	Ret Time	Classification Score	LeftAA	PeptideSequence
1	20211003_Ada5_pE1_D2_2020_pcTPA_NS1_F9	34897	3	True	960.7977	2880.378547	2880.354253	8.4343	5.137	29.933606	0.6494	29	73.68	0.5574856848984951	R	DFVEGVSGGSWVDIVLEHGSCVTTMAK

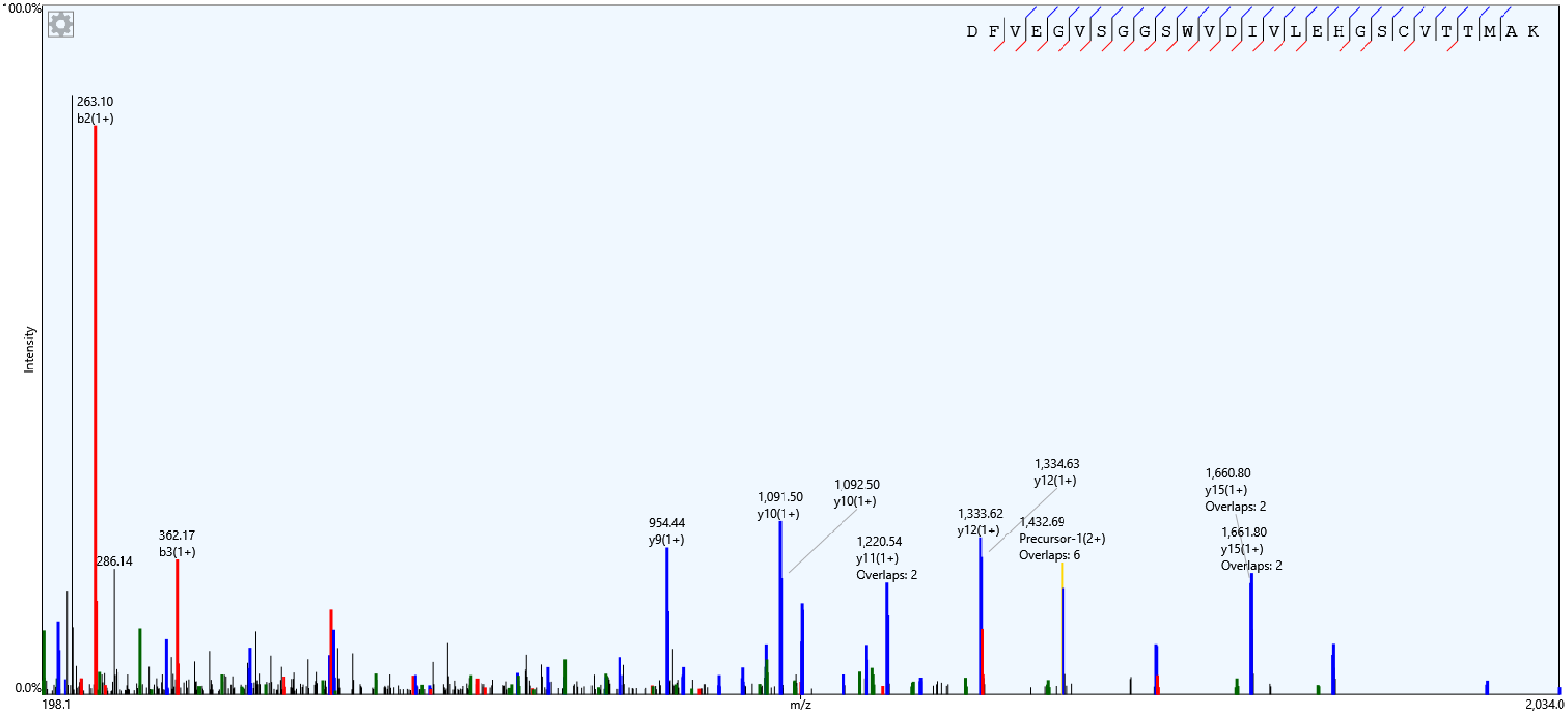

pNS1/E/D2 (fractionsF11&F12): Dengue virus proteins identified (with protein sequence coverage)

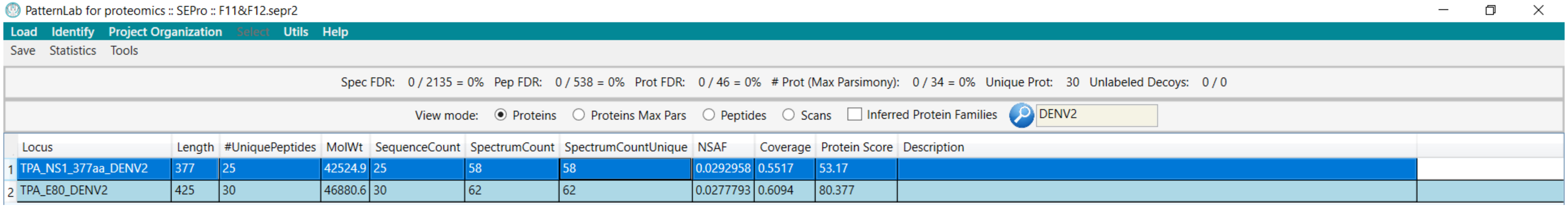

	Locus	Length	#UniquePeptides	MolWt	SequenceCount	SpectrumCount	SpectrumCountUnique	NSAF	Coverage	Protein Score	Description
1	TPA_NS1_377aa_DENV2	377	25	42524.9	25	58	58	0.0292958	0.5517	53.17	
2	TPA_E80_DENV2	425	30	46880.6	30	62	62	0.0277793	0.6094	80.377	

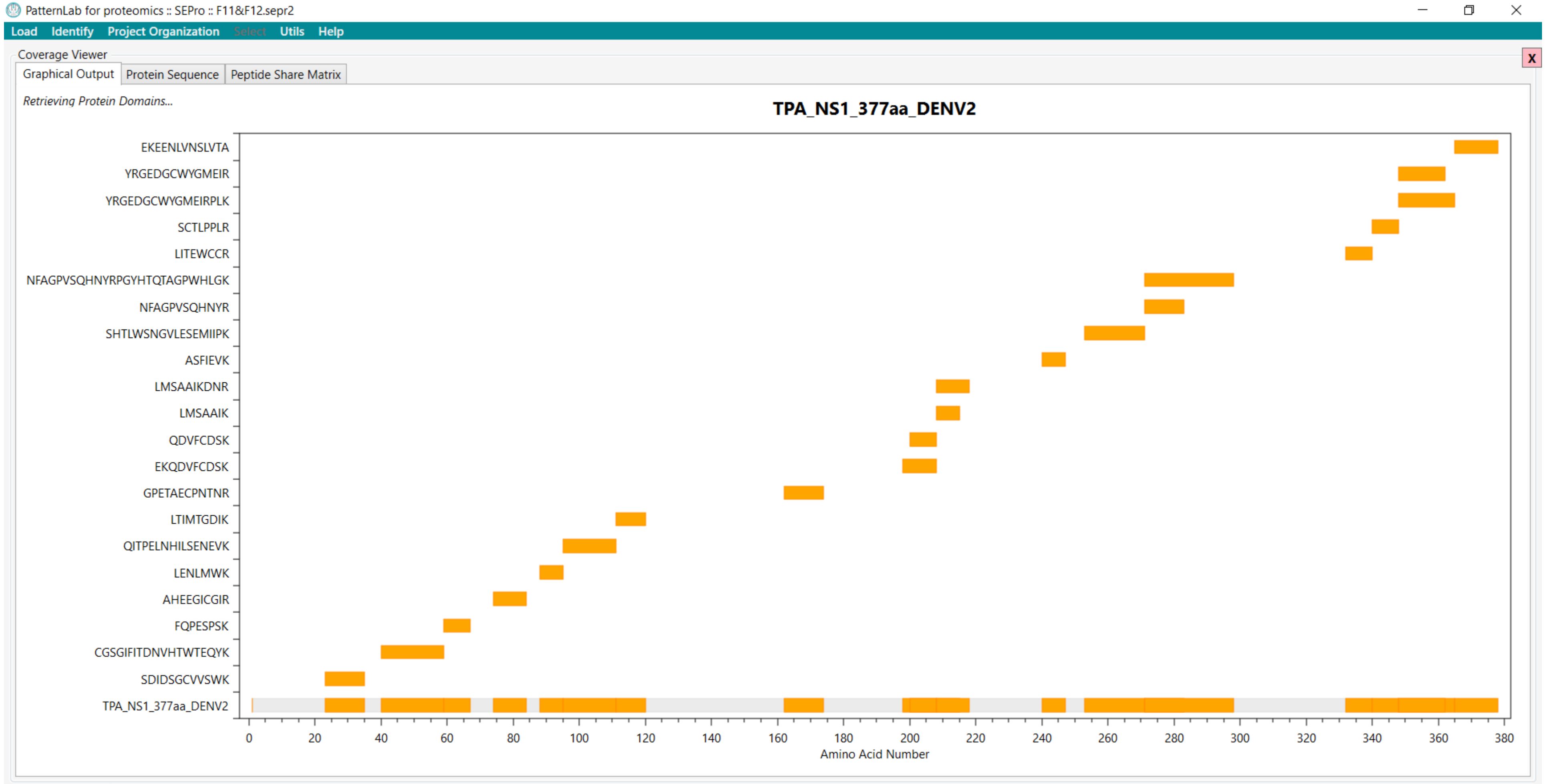

pNS1/E/D2 (fractionsF11&F12): Best MS/MS spectrum identifying protein “TPA_NS1_377aa_DENV2” (top 1 primary score)

	File Name	Scan No	Z	Unique	MZ	MeasuredMH	TheoreticalMH	PPM	PrimaryScore	SecondaryScore	DeltaCN	PeaksMatched	Ret Time	Classification Score	LeftAA	PeptideSequence	RightAA
1	20211003_Ada6_pNS1_E_D2_F12	21061	4	True	537.252	2145.986227	2145.984438	0.8336	3.4243	21.985574	0.7659	22	53.29	0.603054514280753	R	YRGEDGCWYGM[15.9949]EIRPLK	E

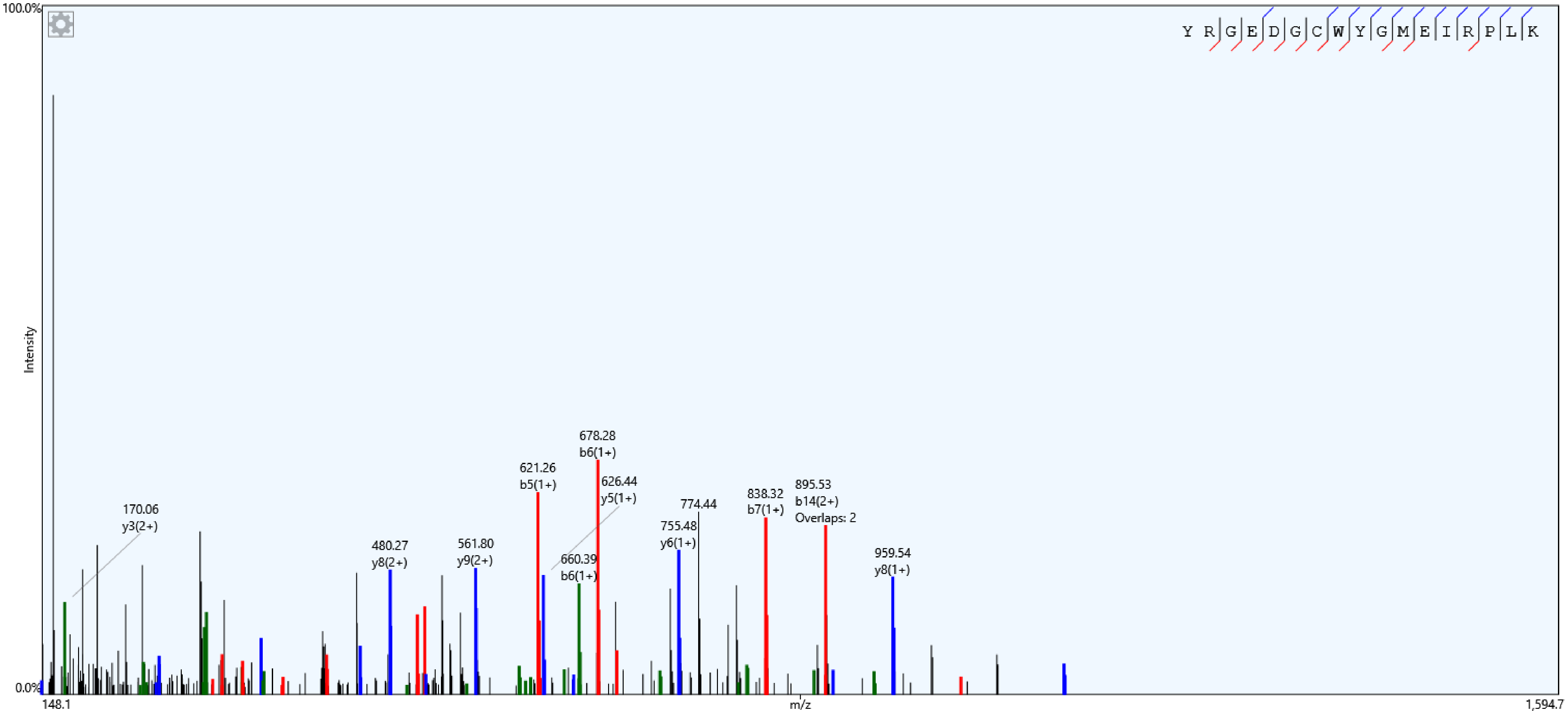

pNS1/E/D2 (fractionsF11&F12): Dengue virus proteins identified (with protein sequence coverage)

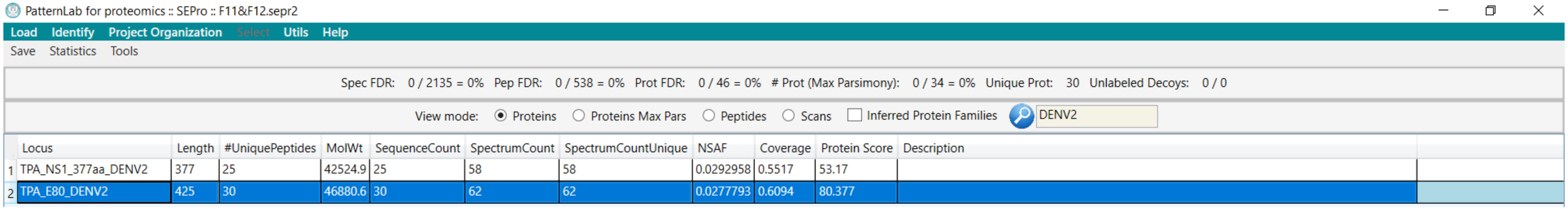

	Locus	Length	#UniquePeptides	MolWt	SequenceCount	SpectrumCount	SpectrumCountUnique	NSAF	Coverage	Protein Score	Description
1	TPA_NS1_377aa_DENV2	377	25	42524.9	25	58	58	0.0292958	0.5517	53.17	
2	TPA_E80_DENV2	425	30	46880.6	30	62	62	0.0277793	0.6094	80.377	

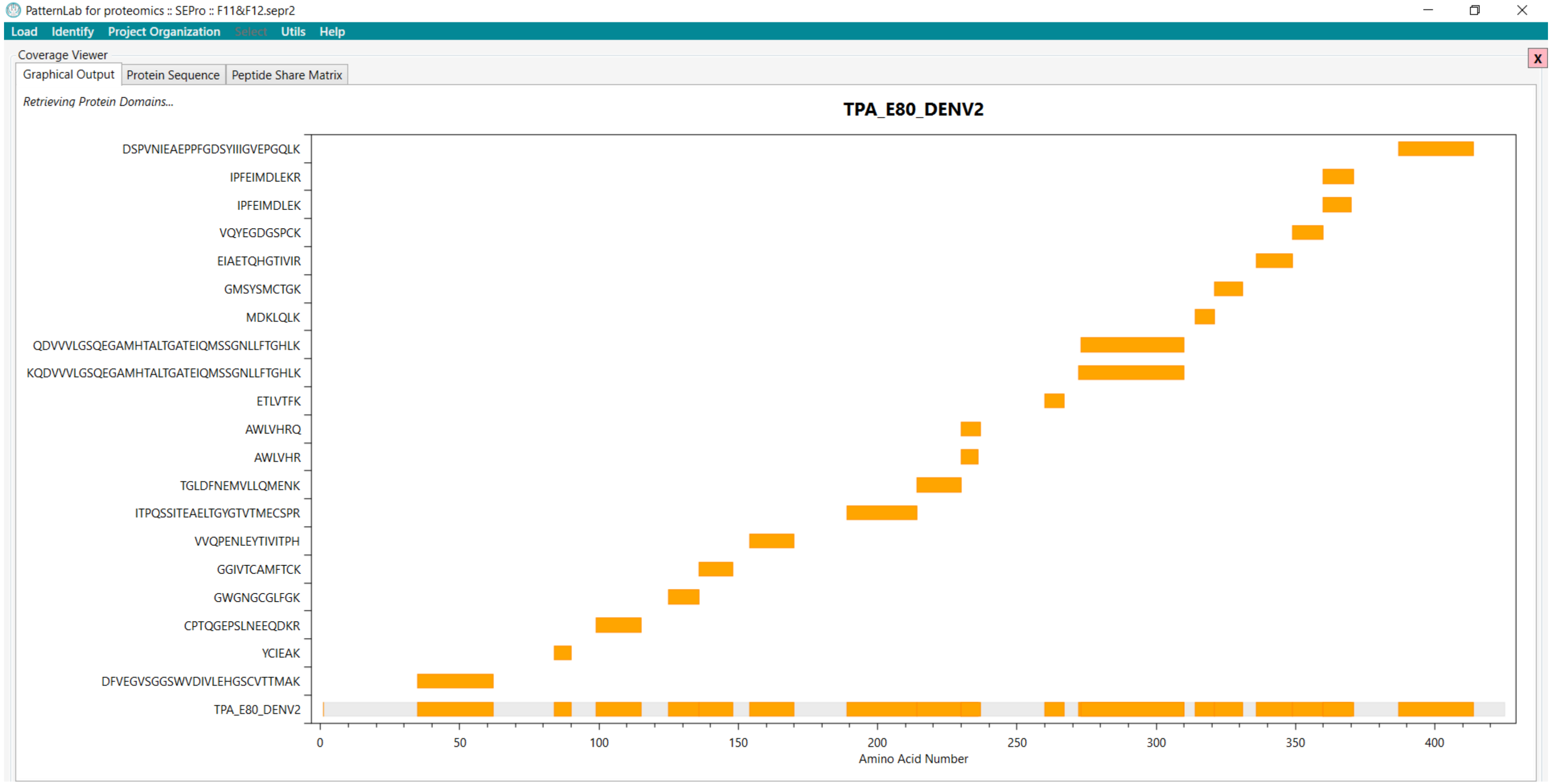

pNS1/E/D2 (fractionsF11&F12): Best MS/MS spectrum identifying protein "TPA_E80_DENV2" (top 1 primary score)

	File Name	Scan No	Z	Unique	MZ	MeasuredMH	TheoreticalMH	PPM	PrimaryScore	SecondaryScore	DeltaCN	PeaksMatched	Ret Time	Classification Score	LeftAA	PeptideSequence	RightAA
1	20211003_Ada6_pNS1_E_D2_F11	31495	5	True	794.612	3969.030894	3969.020849	2.5308	5.7203	37.084708	0.7618	33	69.31	0.5856159556613517	K	KQDVVVLGSQEGAMHTALTGATEIQMSSGNLLFTGHLK	C

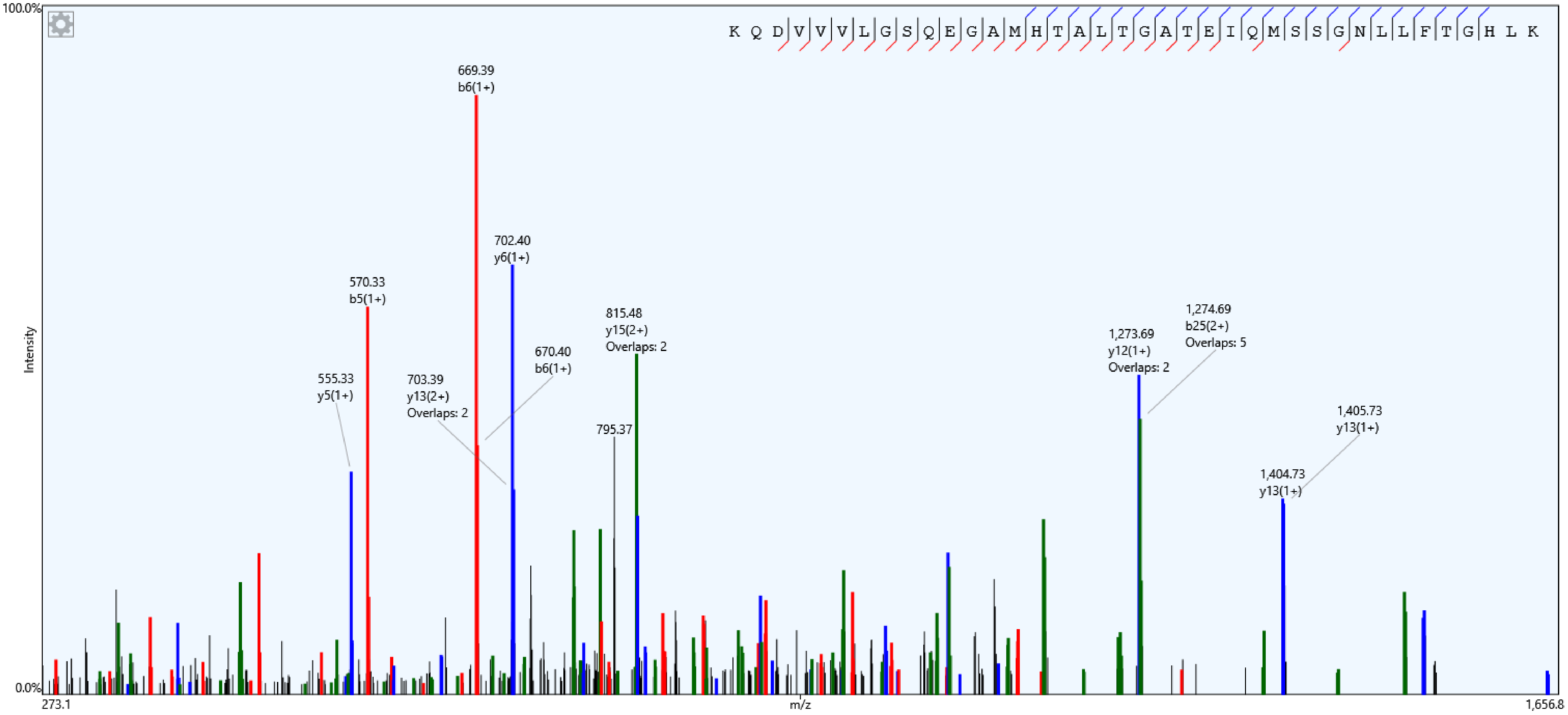

Supplement: Supplementary file 1 [file viruses-14-01452-s001.zip › Figure S4.pdf]
