# Supplementary material for: Combination of E- and NS1-Derived DNA Vaccines: The Immune Response and Protection Elicited in Mice against DENV2
Source: Viruses. 2022 Jun 30;14(7):1452. doi: 10.3390/v14071452 (PMC9323404; doi:10.3390/v14071452)
Supplement: Supplementary file 1 [file viruses-14-01452-s001.zip › Supplementary Material_Legends.pdf]

Combination of E- and NS1-derived DNA vaccines: the immune response and protection elicited in mice against DENV2

Paolla Beatriz A. Pinto [1], Tamiris A. C. Barros [1], Lauro M. Lima [1], Agatha R. Pacheco [1], Maysa L. Assis [1], Bernardo A. S. Pereira [1], Antônio J. S. Gonçalves[1], Adriana S. Azevedo[1], Ana Gisele C. Neves-Ferreira [2], Simone M. Costa [1,*] and Ada M. B. Alves [1,*]

Supplementary Material – Legends

Figure S4: Detailed information on dengue virus proteins identified by MS/MS in the supernatant of BHK-21 cells transfected with different DNA vaccines. For every gel fraction analyzed, we have shown: 1) the protein sequence coverage and all identified peptides; 2) The best MS/MS spectrum identifying each dengue virus protein (top 1 primary score).

Table S1: List of all proteins identified by MS/MS in the supernatant of BHK-21 cells transfected with different DNA vaccines. The results for each plasmid transfection are shown in a different spreadsheet. Identification of NS1 and E proteins were highlighted in green and blue, respectively.
